# DUX4 expression in FSHD muscle cells: how could such a rare protein cause a myopathy?

**DOI:** 10.1111/j.1582-4934.2012.01647.x

**Published:** 2012-12-04

**Authors:** Alexandra Tassin, Dalila Laoudj-Chenivesse, Céline Vanderplanck, Marietta Barro, Sébastien Charron, Eugénie Ansseau, Yi-Wen Chen, Jacques Mercier, Frédérique Coppée, Alexandra Belayew

**Affiliations:** a Laboratory of Molecular Biology, Research Institute for Health Sciences and Technology, University of MonsMons, Belgium; b INSERM U1046 Physiologie et Médecine expérimentale Cœur et MuscleCHU A. de Villeneuve, Montpellier, France; c Children's National Medical Center, Center for Genetic Medicine ResearchWashington, DC, USA

**Keywords:** FSHD, DUX4, homeodomain, differentiation, myoblasts, PITX1, muscle, nucleus

## Abstract

Facioscapulohumeral muscular dystrophy (FSHD) is one of the most frequent hereditary muscle disorders. It is linked to contractions of the *D4Z4* repeat array in 4q35. We have characterized the double homeobox 4 (*DUX4)* gene in *D4Z4* and its mRNA transcribed from the distal *D4Z4* unit to a polyadenylation signal in the flanking *pLAM* region. It encodes a transcription factor expressed in FSHD but not healthy muscle cells which initiates a gene deregulation cascade causing differentiation defects, muscle atrophy and oxidative stress. *PITX1* was the first identified DUX4 target and encodes a transcription factor involved in muscle atrophy. *DUX4* was found expressed in only 1/1000 FSHD myoblasts. We have now shown it was induced upon differentiation and detected in about 1/200 myotube nuclei. The DUX4 and PITX1 proteins presented staining gradients in consecutive myonuclei which suggested a diffusion as known for other muscle nuclear proteins. Both protein half-lifes were regulated by the ubiquitin-proteasome pathway. In addition, we could immunodetect the DUX4 protein in FSHD muscle extracts. As a model, we propose the *DUX4* gene is stochastically activated in a small number of FSHD myonuclei. The resulting mRNAs are translated in the cytoplasm around an activated nucleus and the DUX4 proteins diffuse to adjacent nuclei where they activate target genes such as *PITX1*. The PITX1 protein can further diffuse to additional myonuclei and expand the transcriptional deregulation cascade initiated by DUX4. Together the diffusion and the deregulation cascade would explain how a rare protein could cause the muscle defects observed in FSHD.

## Introduction

Facioscapulohumeral muscular dystrophy (FSHD1A: OMIM #158900) is one of the most common hereditary muscle disorders, affecting seven individuals in 100,000 (http://www.orpha.net), and is associated with contractions of the *D4Z4* repeat array in the 4q35 subtelomeric region [1–3]. In non-affected individuals, this array comprises 11–100 tandem copies of the 3.3-kb *D4Z4* element, whereas in patients with FSHD, only 1–10 *D4Z4* copies are left [1, 3], and at least one *D4Z4* copy is necessary to develop the disorder [4]. A similar DNA hypomethylation associated with an open chromatin structure is observed both on contracted *D4Z4* arrays in FSHD1A and on normal-size arrays in FSHD1B (OMIM #158901) [5–7]. Our group has identified the double homeobox 4 (*DUX4)* gene within each *D4Z4* unit [8] but detection of its mRNA proved very difficult because of its very low abundance and high GC content (discussed in supporting information of [9]). We could identify stable *DUX4* mRNAs in FSHD muscle cells and show that they were transcribed from the most distal *D4Z4* unit of the repeat array and extended to a polyadenylation signal in the flanking *pLAM* region [9]. Those findings were confirmed by other groups which further demonstrated this polyadenylation site was required for *DUX4* mRNA stabilization and to develop FSHD from a contracted allele [10]. The full-length mRNA (*DUX4-fl*) transcribed from this distal *D4Z4* unit contains the entire *DUX4* open-reading frame (ORF) [9, 11, 12]. In addition, a shorter transcript (*DUX4-s*) that might express a protein limited to its double homeodomain was described [11]. The *DUX4-fl* mRNA was detected in most FSHD muscle cells and biopsies, whereas the *DUX4-s* mRNA was detected both in healthy control and some FSHD samples [11]. The *DUX4-fl* mRNA was also detected in FSHD1B muscle cells [11]. In addition, we have characterized a *DUX4* homologue mapped 42 kb centromeric of the *D4Z4* repeat array and named *DUX4c*. The encoded protein is expressed in healthy muscle cells and induced in FSHD [13]. Because it activates myoblast proliferation and inhibits their differentiation, DUX4c might be involved in muscle regeneration, and changes in its expression could contribute to the FSHD pathology [13–15]. In aggregate, our discovery of the functional *DUX4* and *DUX4c* genes in repeated DNA elements has contributed to the obsolescence of the ‘junk DNA’ concept [16].

Detection of the DUX4 protein proved a technical challenge because of its particularly low abundance. Our initial detection in primary FSHD but not control myoblast cultures [9] was confirmed by Snider *et al*. who found it was in fact expressed at a relatively abundant level in very few nuclei (1/1000 myoblasts) [11]. DUX4 overexpression in cell cultures led to cell death [17]. Intriguingly, myotubes, but not myoblasts, were somehow protected against DUX4-induced cell death [18–20]. The DUX4 protein is a transcription factor that targets a large set of genes, some of which encode other transcription factors that in turn target additional genes [9, 18, 19, 21]. Indeed, DUX4 directly activates the *PITX1* (Paired-like homeodomain transcription factor 1) gene which is specifically induced 10- to 15-fold in FSHD muscles as compared with 11 other neuromuscular disorders [9]. The PITX1 protein itself is a homeodomain transcription factor involved in hindlimb identity specification during embryogenesis, and can induce adult muscle atrophy [22]. A large number of genes were identified in the deregulation cascade caused by DUX4 overexpression either in mouse C2C12 cells [18] or human primary myoblasts [21]. DUX4 expression in myoblast cultures recapitulated key features of the FSHD molecular phenotype, *i.e*. repression of MyoD and its target genes leading to diminished myogenic differentiation, repression of glutathione oxydo-reduction pathway components resulting in increased sensitivity to oxidative stress, muscle atrophy and activation of germline-specific genes [18, 19, 21]. We have recently demonstrated the DUX4 causal role in the atrophy process by gain and loss of function experiments in primary human myoblasts followed by differentiation to myotubes. DUX4 overexpression induced hypomorphic myotube formation associated with the induction of E3 ubiquitin ligases (MURF1 and Atrogin1) typical of muscle atrophy, whereas RNA interference or antisense oligonucleotides targeting the DUX4 mRNA reversed this phenotype [16]. In addition, DUX4 overexpression in mouse muscles *in vivo* caused a TP53-dependent myopathy requiring the DUX4 DNA binding domain [23]. In aggregate, these studies confirmed the major role played by DUX4 in the pathological mechanism of FSHD through the initiation of a large transcription deregulation cascade (reviewed in [20]). The question that still remained unclear was how such a scarce protein could lead to the muscle pathology in FSHD.

In the present study, we focused on DUX4 protein expression and degradation in FSHD myotubes. Barro *et al*. have established a panel of primary CD56^+^ myoblasts derived from patients with FSHD and matched healthy individuals (controls). These FSHD myoblasts fused and differentiated into myotubes with morphological abnormalities: they were either thin ‘atrophic’ myotubes or disorganized ones with clusters of non-aligned nuclei. Both phenotypes were found in different proportions in each myotube culture derived from a patient with FSHD. A myotube is a multinucleated syncitium in which nuclei share a large cytoplasm. Both muscle-specific or housekeeping genes are transcribed in stochastic pulses that occur independently in individual myonuclei [25]. The mRNAs expressed from a given myonucleus are translated in its adjacent cytoplasmic domain, and if the synthesized proteins carry a nuclear localization signal (NLS), they are imported into this active nucleus. As initially shown with a beta-galactosidase protein fused to a NLS, these proteins can also be imported at a reduced level into nuclei on either side of the source of expression [26]. This diffusion phenomenon was confirmed by several research groups [25, 27, 28], but was never described in pathological myotubes. In this study we have investigated DUX4 and PITX1 expression in myotubes from affected and non-affected muscles of patients with different age or gender. Our results suggest that as described for other muscle transcription factors, the DUX4 protein appears in a single nucleus where the gene is likely stochastically activated and diffuses to adjacent myonuclei. We propose that this DUX4 expression pulse initiates a transcriptional amplification cascade [19] that progressively extends in consecutive myonuclei to the whole myotube causing the FSHD pathological phenotype.

## Materials and methods

### Muscle biopsies and ethics statement

Primary human myoblasts, muscle biopsies and surgical muscle surplus during scapular fixation were obtained according to procedures approved by the current ethical and legislative rules of France or Belgium and written informed consent was obtained from all participants, as directed by the ethical committee of CHU de Villeneuve (Montpellier, France) [24]. In addition, the uses of this material have been approved by the ethics committee of the University of Mons (ref #A901). We used clinical and histopathology criteria as described [24] to assess whether the biopsied muscle was affected and to evaluate the severity.

### Cell Culture

C2C12 (mouse myoblasts) and TE671 (human rhabdomyosarcoma) cells were grown in DMEM high glucose (4.5 g/l) with L-glutamine and sodium pyruvate (PAA Laboratories GmbH, Pasching, Austria), 1% antibiotic/antimycotic (PAA Laboratories) and 10% foetal calf serum (FCS; PAA Laboratories) at 37°C under 5% CO_2_. These cells were transfected with the *pCIneo-DUX4* expression vector to provide a positive control for Western blots (Figs 5C, 6A, Figs S2A, S3 and S6) or with the empty *pCIneo* vector as a negative control (Fig. S3A). For transfections, C2C12 cells (5 × 10^5^) were seeded in a 75 cm^2^ flask and transfected 24 hrs later in Opti-MEM (Invitrogen, Carlsbad, CA, USA) with 20 μg plasmid and 60 μl Lipofectamin2000 (Invitrogen). TE671 cells (1.2 × 10^6^) were seeded in a 75 cm^2^ flask and transfected 24 hrs later in their culture medium with 10 μg plasmid and 32 μl FuGENE6 (Roche Diagnostics, GmbH, Mannheim, Germany). Cells were harvested 24 hrs post-transfection.

**Fig. 1:**
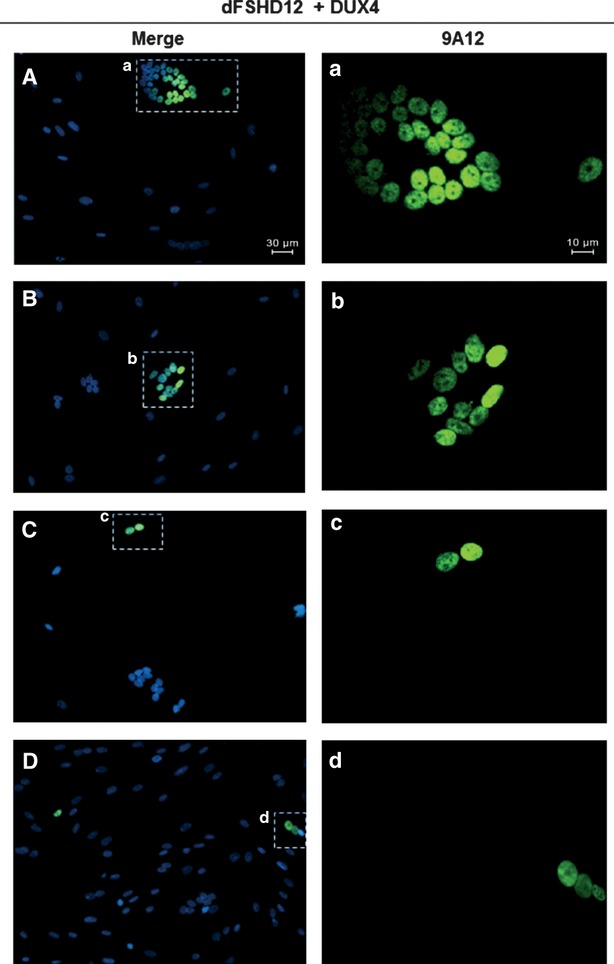
Overexpressed DUX4 is detected in consecutive myonuclei of individual myotubes. Facioscapulohumeral muscular dystrophy (FSHD) myoblasts (dFSHD12) were transfected with the *pCIneo-DUX4* expression vector at a low efficiency and differentiation was induced 5 hrs later. DUX4 (green) was detected by immunofluorescence with the MAb 9A12 monoclonal antibody 3 days after transfection. DUX4 was detected in myotubes containing either clusters of nuclei (**A** and **B**) or aligned nuclei (**C** and **D**). The nuclei were visualized by DAPI staining (blue). a, b, c and d correspond to enlarged fields from the left boxes.

Primary myoblast cultures from control individuals and patients with FSHD were isolated from muscle biopsies, purified by a selection of CD56^+^ cells and established as described [24]. They were grown in collagen-coated dishes (Iwaki Cell Biology, Tokyo, Japan) in DMEM with high glucose (4.5 g/l), sodium pyruvate and sodium bicarbonate (Sigma-Aldrich, St Louis, MO, USA) with L-glutamine (4 mM; Sigma-Aldrich), Gentamycin (50 μg/ml; Sigma-Aldrich), 10% foetal bovine serum (FBS; Invitrogen) and 1% Ultroser G (Pall BioSepra, Cergy-St-Christophe, France) at 37°C under 5% CO_2_. Before experimentation, primary myoblasts were seeded in 10 cm or 35 mm collagen-coated dishes, respectively, for Western blot or immunofluorescence, in DMEM with Gentamycin (50 μg/ml) and 20% FBS. The myogenic differentiation of confluent cells was induced by decreasing the FBS concentration to 2%. The proteasome inhibitor MG132 (25 μM or 50 μM; Sigma-Aldrich) was added to the culture medium 5 hrs before harvesting the cells (Fig. 5). For transfection, primary myoblasts were seeded in the growth medium (DMEM/Gentamycin/10% FBS/1% Ultroser G) and transfected 24 hrs after seeding with Fugene HD (Roche Diagnosis) and plasmid DNA at a 6:2 ratio as described [19]. Myoblasts were then differentiated 5 hrs later by replacing the medium to DMEM/Gentamicin/2% FBS during 3 days (Fig. 1). A ‘reverse transfection’ with SiPORTNeoFX transfection agent (Applied Biosystems, Ambion, Austin, TX, USA) was used to introduce short interfering (si)RNA against *DUX4* or *DUX4c* (Fig. S2) in primary myoblasts as described in [19]. Differentiation was induced 5 hrs later as above and cells were fixed for immunofluorescence after 3 days.

### Plasmid constructs

The *pCIneo-DUX4* and *pCIneo-DUX4c* expression plasmids were described previously in [8, 9, 13] and contain the respective ORF under control of the CMV promoter. The vector used to determine the transfection efficiency by monitoring of green fluorescence was the *pEGFP-N2* (Clontech, Mountain View, CA, USA).

### DUX4 and DUX4c antibodies

The mouse monoclonal antibody against DUX4 (MAb 9A12) was raised against the 253 last residues of the DUX4 carboxyl-terminal domain as described in [9]. The anti-PITX1 rabbit serum was raised against a PITX1-specific peptide as described in [9]. The anti-DUX4c rabbit serum was raised against a carboxyl-terminal peptide as described in [13]. The 314 rabbit antiserum was raised against a DUX4-specific peptide corresponding to residues 342–356 as described in [17].

### Immunodetection by Western blot

The following protocol was specifically developed for the detection of the endogenous DUX4 protein with MAb 9A12. Whole-cell extracts of myoblast primary cultures were obtained by lysis in hypertonic buffer (50 mM Tris pH 7.0, 50 mM NaCl, 0.1% Nonidet P40, 1 mM DTT) and protease inhibitor cocktail (Sigma-Aldrich) using three freeze/thaw cycles. Nuclear extracts were prepared with the NE-PER Nuclear and Cytoplasmic Extraction Reagent kit (Thermo Scientific, Rockford, IL, USA) according to the manufacturer's procedure except that nuclei were lysed with the hypertonic buffer. Forty μg cell lysate or 20 μg nuclear extract were separated by 10 or 12% PAGE-SDS during 3 to 4 hrs at 100 V and electrotransferred onto a nitrocellulose membrane (GE Healthcare Europe GmbH, Diegem, Belgium). The electrotransfert was performed at 4°C in a wet tank with a blotting buffer containing 12.5 mM TRIS, 192 mM Glycine and 20% Methanol, at 160 mA during 90 min. The membrane was stained with Ponceau red to check loading and migration quality, and a picture was taken for loading control. After rinsing in PBS, the Western blot was blocked 1 hr at room temperature in phosphate buffered saline (PBS) with 5% non-fat dry milk, rinsed in PBS and incubated overnight at 4°C with MAb 9A12 (1:1000) in PBS-BSA 2%. After rinsing in PBS, appropriate secondary antibodies coupled to HRP (1:5000; GE Healthcare) were added and detected with the super signal west femto maximum sensitivity substrate (Thermo Scientific) on Amersham Hyperfilm ECL (GE Healthcare).

For immunodetection on muscle biopsy extracts (Fig. 6A and Fig. S6), the same protocol was used with the homogenization in the hypertonic buffer. In Figure 6A, 40 μg (F11 and F7) or 80 μg (F10 and C1) protein extract were loaded. In Figure S6, 40 μg of each protein extract was loaded. For the 2D gel analysis, an isoelectrofocalization (IEF) was performed with the IPGphor system (Amersham Pharmacia Biotech) according to the manufacturer's instructions, with modifications as described [29]. Immobiline Dry Strip (pI 3–11), IPG buffer pH 3–11 and electrophoretic reagents were purchased from Amersham Pharmacia. The second dimension was a PAGE-SDS.

### Immunofluorescence

Primary myoblasts seeded on 35 mm collagen-coated dishes (Iwaki Cell Biology) were fixed 5 min. at room temperature (RT) in 4% paraformaldehyde. Cells permeabilization was performed in PBS 0.5% Triton X-100, 5 min. at RT. After blocking in PBS 20% FBS, cells were incubated with primary antibodies during 2 hrs at RT. The following antibodies and dilutions were used: MAb 9A12 (purified: 1/50 or hybridoma culture medium: 1:1), anti-PITX1 rabbit serum (1/50), anti-DUX4c rabbit serum (1/50), rabbit polyclonal antidesmin ab15200 (1/200; Abcam, Cambridge, UK), rabbit MAb anticleaved PARP (1/200, overnight at 4°C; Cell Signaling, Danvers, MA, USA). After washing and blocking, cells were incubated during 1 hr at RT with Alexa Fluor secondary antibodies goat antimouse 488 and antirabbit 555 (1/100; Invitrogen). After washing, cells were covered by 5 μl of Vectashield mounting medium (Vector Laboratories, Burlingame, CA, USA) containing 4,6-diamidino-2-phenylindole (DAPI) and by a coverslip.

The detection of DNA fragmentation in DUX4-positive nuclei was performed with the Apoptag Red *In Situ* Apoptosis Detection kit (Millipore, Billerica, MA, USA), according to the manufacturer's instructions.

### Microscopy

Microscopy images were collected with the following workstations: the Montpellier RIO Imaging facility at the CRBM, Montpellier, France (http://www.mri.cnrs.fr); the imagery platform at the IBMM, ULB, Belgium (http://www.cmmi.be) and a Nikon Microscope Eclipse 80i with a DS-U3 DS Camera control Unit and the NIS element-BR analysis software. Plan Fluor 20 X, Plan Fluor 40× and 60× Apo-VC high-resolution oil immersion objectives were used with 350-, 480- and 540- nm excitation for the 4,6-diamidino-2-phenylindole (DAPI), fluorescein isothiocyanate (FITC) and tetramethylrhodamine isothiocyanate (TRITC) channel respectively.

### Quantifications and Statistics

**Fig. 2:**
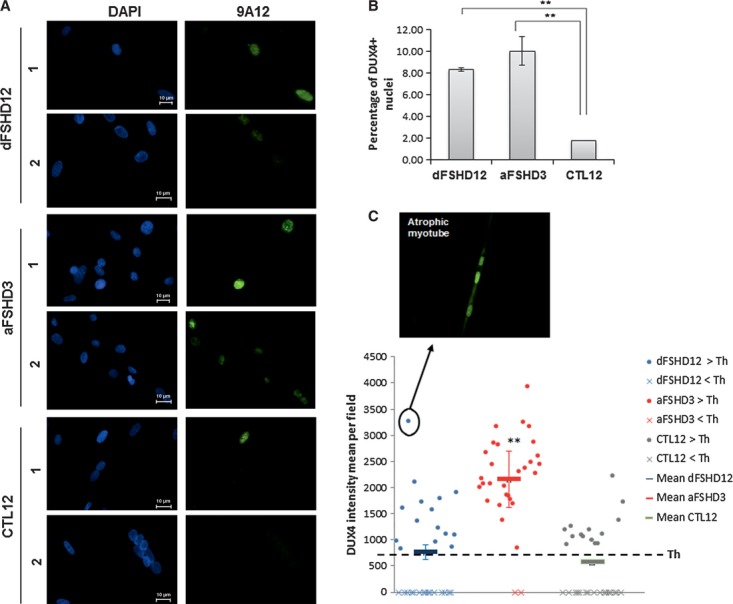
Endogenous DUX4 expression in facioscapulohumeral muscular dystrophy (FSHD) and control primary myotubes. (**A**) DUX4 (green) was detected by immunofluorescence with MAb 9A12 in the nuclei of disorganized (dFSHD12) and atrophic (aFSHD3) FSHD or control (CTL12) myoblasts 4 days after the induction of differentiation. DAPI (blue) was used to visualize nuclei. (**B**) Quantification of DUX4-positive nuclei in aFSHD3 and dFSHD12 myotubes compared with control (CTL12) myotubes 4 days after the induction of differentiation. The number of DUX4-positive nuclei was counted in 30 random fields of three independent experiments (10 fields per experiment). Two representative fields (1, 2) used for the quantification are shown in (A) for each cell line. The percentage was calculated relatively to the number of DAPI-positive nuclei, and the histogram represents the percentage means. The significance was evaluated by an anova test. **P < 0.01 was considered significant. (**C**) Quantification of DUX4-positive nuclei intensity in aFSHD3 and dFSHD12 myotubes compared with control (CTL12) myotubes 4 days after the induction of differentiation. The intensity of DUX4-positive nuclei was measured in 30 random fields of three independent experiments. Intensity values below the threshold (Th) are considered as null. Each value was plotted. Rectangles represent the intensity means. The significance was evaluated by an anova test. **P < 0.01 was considered significant.

The number of DUX4-positive nuclei (Fig. 2B and Fig. S2C) was counted from at least 10 random fields (20× objective). The percentage was calculated relatively to the number of DAPI-positive nuclei per field and the histograms represent the percentage mean. The DUX4 intensity mean per field (Fig. 2C) was measured using the NIS element-BR analysis software. Intensity values below the threshold (Th: corresponding to DUX4 intensity mean in disorganized culture) are considered as null.

The significance was evaluated by an anova test and a multiple comparison of means (Tukey Contrasts) using the ‘R Foundation for Statistical Computing 2.14.0’. An arcsine transformation (*p*′ = arcsin√*p* where *p* = proportion), commonly used for proportions, was applied beforehand to the data. **P < 0.01 was considered significant.

## Results

### Exogenous DUX4 proteins are detected in consecutive nuclei of individual myotubes

In a first experiment, we wanted to optimize immunostaining conditions that would allow DUX4 detection in very few myonuclei. We used an FSHD primary myoblast line (dFSHD12) that forms mostly disorganized myotubes but also some atrophic ones [24]. We transfected these cells at the myoblast stage with a *DUX4* or *EGFP* expression vector at a very low efficiency and induced fusion into myotubes 5 hrs later. The immunofluorescence was performed 3 days later with MAb 9A12, a monoclonal antibody we had previously developed against the DUX4 carboxy-terminal region [9].

As expected, only 12% and 16% of the transfected cells expressed high levels of EGFP or DUX4 respectively (data not shown). About 58% of EGFP-expressing nuclei were present in myoblasts and 42% in myotubes. Surprisingly, the majority (88%) of the DUX4-positive nuclei were found in pluri-nucleated cells (myotubes) and only 12% in cells containing a single nucleus (myoblasts). The DUX4 immunostaining followed an intensity gradient in consecutive nuclei of a given myotube (Fig. 1). DUX4 was detected either in disorganized myotubes containing myonuclei clusters (>10 nuclei) (Fig. 1A and B) or in atrophic myotubes with a small number of aligned nuclei (from two to eight nuclei) (Fig. 1C and D). A similar staining pattern was observed using a rabbit serum directed against a DUX4-specific peptide (data not shown, Ab #314; [17]). In these experiments, when DUX4 was found in at least one myotube nucleus, the consecutive myonuclei were often also DUX4 positive. These intensity gradients suggested that the *DUX4* mRNA transcribed in one myonucleus was translated in the adjacent cytoplasm domain into proteins that could be imported in several neighbouring nuclei, a diffusion mechanism previously described for other muscle proteins [25–28, 30].

### The endogenous DUX4 protein is expressed in differentiating FSHD myoblasts

We have previously immunodetected the DUX4 protein with MAb 9A12 in primary cell cultures derived from FSHD muscles but not from healthy controls [9]. We have now further demonstrated this antibody specificity (see Figure S1, and RNA interference experiment Figure S2) and used it to detect DUX4 on Western blots prepared with three additional FSHD and two control primary myoblast lines. DUX4 was detected not only in extracts of confluent myoblasts confirming our previous data [24] but also in differentiated myotubes (Fig. S3). Intriguingly, we detected a very weak signal in one control sample (CTL3 diff) prepared from cells grown 6 days in the differentiation medium (Fig. S3A).

DUX4 was detected in differentiated FSHD primary myoblast cultures whether they presented a higher proportion of either disorganized or atrophic myotubes (Fig. 2, Table 1). The DUX4-positive nuclei were counted in triplicate in 10 random fields and found significantly higher in the atrophic and disorganized FSHD myotube cultures than in the controls (Fig. 2B, **P < 0.01). The number of nuclei with a strong DUX4 immunofluorescence was the highest in the atrophic FSHD cultures (**P < 0.01). The average of DUX4 nuclear staining intensity was about threefold lower and similar in control and disorganized FSHD cultures, except for a single atrophic myotube found in the latter (Fig. 2C). The DUX4 expression level differed in individual FSHD primary myoblast lines with no correlation with the non-affected or affected (*) status of the muscle it had been derived from (Figs S1, S3 and Table 1). All the data concerning the FSHD and control primary myoblast lines used in this study (muscle type, myotube phenotype, etc.) are summarized in Table 1 and Table S1 respectively.

**Table 1:** Summary of data about DUX4 expression in FSHD primary myoblasts. Name of the FSHD cell line (NB: ‘line’ refers to the myoblast population derived from a single biopsy); age and gender of the patient (M: male; F: female); number of *D4Z4* units; site of the muscle biopsy (T: *trapezius*; Q: quadriceps (*vastus lateralis*); FB: femoral biceps); score on the Brooke–Vignos scale defining the clinical status of upper and lower limb muscles, respectively, where high values define affected muscles and low values define non-affected muscles [44, 45]; predominant phenotype of the derived myotubes and MFI (myoblasts fusion index: ratio between the nuclei present in myotubes *versus* the total number of nuclei in a given microscope field; the proportion of atrophied myotubes in a culture is inversely correlated with the MFI). DUX4 protein detection is indicated (+) as well as the number of days in differentiation medium (diff) and the method used (WB: immunodetection on Western blot; IF: immunofluorescence). The information about the patients and the morphological characteristics of each FSHD cell line was previously determined in [24]

Code#	Age	Gender	*D4Z4* units	Muscle type (Brooke-Vignos scale)	Myotubes phenotype (MFI)	DUX4 expression (Methods)	Differentiation state (Figures)
aFSHD1*	30	M	5	T(4-5)	Atrophic (18%)	++(IF, WB)	-WB: aligned myoblasts (Fig. S3A)-IF: diff 4 (Figs 4B, 5B, S1A)
aFSHD3	32	F	7	Q(1-1)	Atrophic (37%)	+(IF,WB)	-WB: diff 3 (Fig. S2A); diff 4 (Fig. 5C)-IF: diff 4 (Figs 2A, 4E, S4A)
aFSHD5	53	M	6	Q(2-3)	Atrophic (42%)	+(WB)	Diff 4 and diff 8 (Fig. S3B)
a/dFSHD7	53	M	9	FB(2-2)	Atrophic (49%)	+(WB)	Aligned myoblasts (Fig. S3A)
dFSHD12	38	F	7	Q(1-1)	Disorganized (60%)	+(IF, WB)	-WB: diff 4 (Fig. S3B)-IF: diff 4 (Figs 1, 2A, 4C/D, 5B, S1B, S4A-C); diff 5 (Fig. 3)
dFSHD13*	42	F	8	Q(4-4)	Disorganized (70%)	+(IF)	-IF: diff 4 (Figs 4A, 5A, S1B)

# All these samples were characterized in Barro *et al*., [24].

* myoblasts derived from affected muscle.

### The endogenous DUX4 protein is expressed in myoblasts and in consecutive myotube nuclei

**Fig. 3:**
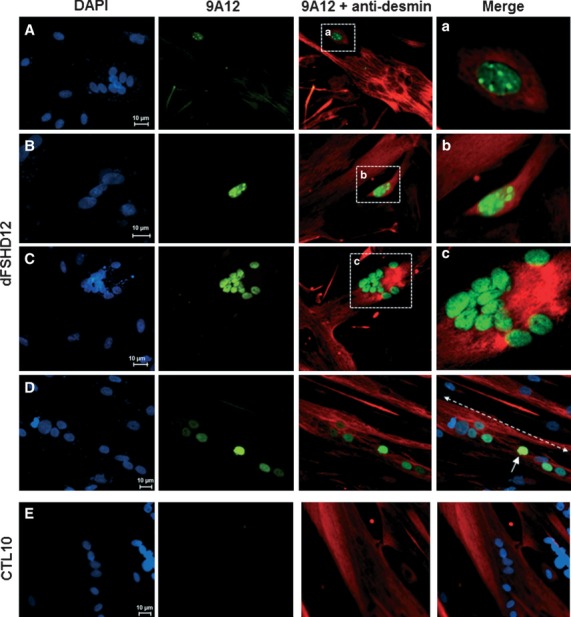
DUX4 is expressed in facioscapulohumeral muscular dystrophy (FSHD) myoblasts and in consecutive nuclei in FSHD myotubes. Co-immunofluorescence with MAb 9A12 (green) and a rabbit serum directed against desmin (red) on FSHD (dFSHD12) and control (CTL10) primary myotubes, 5 days after the induction of differentiation. a, b and c correspond to enlarged fields from the left boxes. Arrows indicate the most stained nuclei and the dotted arrows the intensity gradient of the DUX4 staining (D: merge panel). DAPI (blue) was used to visualize nuclei.

A co-immunostaining was performed with MAb 9A12 and a rabbit serum directed against desmin to determine whether DUX4-positive nuclei belonged to either isolated myoblasts or myotubes. A total of 5% of the nuclei stained for DUX4 among which 16% were in non-fused myoblasts found in the vicinity of myotubes. Among these DUX4-positive myoblasts, 77% presented the staining as nuclear foci (Fig. 3A and B). Again, when DUX4 was detected in at least one myotube nucleus, it also was in the adjacent myonuclei. In myotubes, the DUX4 staining appeared either in clusters of nuclei with various intensities (Fig. 3C) or in aligned nuclei with a clear intensity gradient (Fig. 3D). Some DUX4-positive nuclei presented an abnormal morphology that might reflect its toxicity. However, no overt apoptosis markers could be detected in these nuclei (Fig. S4).

### DUX4 and PITX1 are either coexpressed or expressed in distinct nuclei of FSHD myotubes

**Fig. 4:**
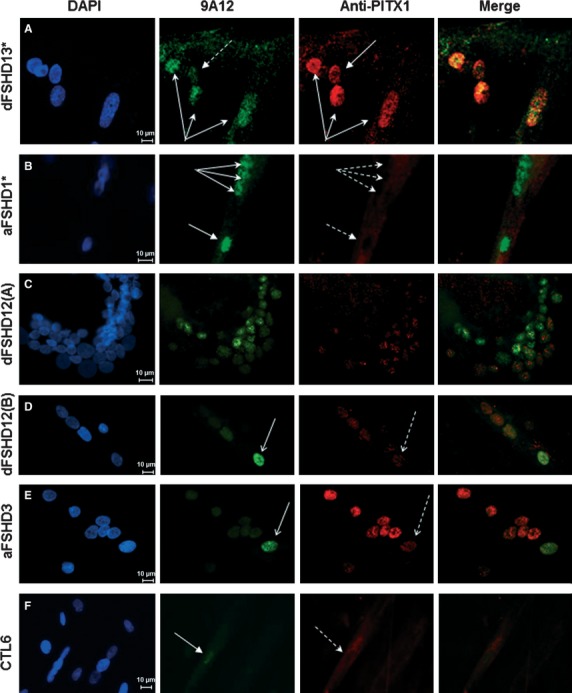
DUX4 and PITX1 detections in facioscapulohumeral muscular dystrophy (FSHD) myotubes. DUX4 (green) and PITX1 (red) were detected by immunofluorescence with MAb 9A12 or the rabbit anti-PITX1 serum in nuclei of primary myotubes 4 days after the induction of differentiation. Colocalization of DUX4 and PITX1 staining appears yellow (Merge). DAPI (blue) was used to visualize nuclei. dFSHD13 and aFSHD1 are derived from an affected muscle (*) and dFSHD12 and aFSHD3 from a non-affected muscle. Arrows indicate positive nuclei for DUX4 or PITX1 staining, and dotted arrows indicate PITX1 or DUX4-negative nuclei.

We have previously shown that DUX4 overexpression induced the endogenous *Pitx1* gene in mouse C2C12 cells and that the induced Pitx1 nuclear protein colocalized with DUX4 [9]. In the present study, we detected the endogenous DUX4 and PITX1 proteins in human FSHD primary myotubes by a co-immunofluorescence using MAb 9A12 and a rabbit serum against PITX1 [9] (Fig. 4). About 4% of the nuclei were positive for DUX4 in both atrophic (Fig. 4B and E) and disorganized FSHD myotubes (Fig. 4A, C and D). PITX1 staining was found in 5% of the nuclei either in the DUX4-positive nuclei with partial colocalization (1%) (Fig. 4A, merge pictures) or in different nuclei (4%) (Fig. 4B–E, merge pictures and arrows). Like DUX4, PITX1 was often detected in consecutive myonuclei (Fig. 4A, C–E). In a given myotube, the PITX1-positive nuclei were often localized close to a DUX4-positive nucleus (Fig. 4D and E).

### The DUX4 and PITX1 protein half-lifes are regulated by the proteasome

**Fig. 5:**
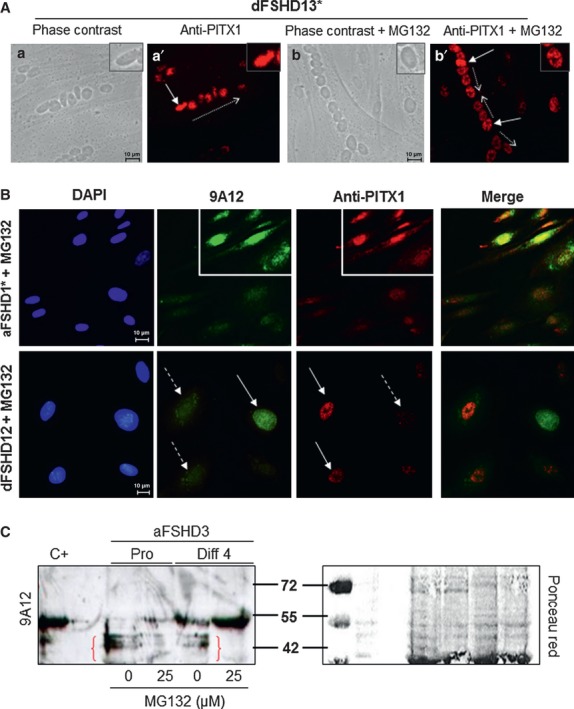
DUX4 and PITX1 stabilization in facioscapulohumeral muscular dystrophy (FSHD) myotubes treated with MG132. (**A**) PITX1 (red) was detected by immunofluorescence with the rabbit anti-PITX1 serum in nuclei of myotubes 4 days after the induction of differentiation (a', b'). Phase contrast microscopy was used to visualize the myotube morphology and position of the nuclei (a, b). b and b' correspond to primary myotubes treated with 25 μM MG132, a proteasome inhibitor, for the last 5 hrs in culture before fixation. Arrows indicate the most stained nuclei and the dotted arrows the intensity gradient of PITX1 staining. (**B**) DUX4 (green) and PITX1 (red) were detected by immunofluorescence with MAb 9A12 or the rabbit anti-PITX1 serum, respectively, in nuclei of FSHD (aFSHD1 and dFSHD12) primary myotubes 4 days after the induction of differentiation. The myotubes were treated with 25 μM MG132 as in (A). Colocalization of DUX4 and PITX1 staining appears yellow (Merge). DAPI (blue) was used to localize nuclei. Arrows indicate positive nuclei for DUX4 and PITX1 staining and dotted arrows indicate negative nuclei for DUX4 and PITX1 staining. (**C**) Nuclear proteins extracted from aFSHD3 primary myoblast were analysed by 12% PAGE-SDS followed by Western blotting and immunodetection with MAb 9A12 as described in Materials and Methods. Myotubes were treated with 0 or 25 μM MG132 as indicated 5 hrs before harvest. Nuclear extracts were prepared using the NE-PER kit at the proliferation state (pro) or 4 days after the induction of differentiation (diff 4). Total extracts of TE671 cells transfected with *pCIneo-DUX4* were used as a positive control (C^+^). DUX4 proteolysis products observed in the absence of MG132 are shown by red braces. Ponceau red staining of the membrane was used as loading control.

The staining patterns we observed above for DUX4 and the product of its *PITX1* target gene suggested a dynamic expression regulated by proteolysis. As previously mentioned [9] and according to the PESTfind software (http://emboss.bioinformatics.nl/cgi-bin/emboss/epestfind), the PITX1 protein harbours a PEST motif (Fig. S5A), *i.e*. a sequence enriched in proline (P), glutamic acid (E), serine (S) and threonine (T) that targets proteins for rapid degradation by the proteasome [31]. Although PITX1 was immunodetected in standard conditions (Figs. 4 and 5Aa'), we could improve its labelling by addition of MG132 to the culture medium for 5 hrs prior to cell harvest. In the presence of this proteasome inhibitor, PITX1 presented a different intranuclear staining pattern (Fig. 5Ab' and B, enlarged inset). Interestingly, as was observed for DUX4, some nuclei presented a strong PITX1 labelling that progressively decreased in the consecutive nuclei (Fig. 5Ab'). These data suggested a gradual diffusion within a given myotube of both DUX4 and PITX1 proteins in nuclei adjacent to an initial single nucleus where their gene had been activated. As mentioned above, we observed either partial DUX4/PITX1 colocalization or mutually exclusive labelling (Fig. 5B, arrows and dotted arrows).

DUX4 detection was improved by MG132 addition even though it only displayed poorly predicted PEST motifs in its carboxyl-terminal domain. However, the PEST score (a combination of enrichment in D, E, P, S and T residues and hydrophobicity) of some DUX4 residues was just below the threshold score (5.0) (Fig. S5B). In addition, according to the Protparam software (http://www.expasy.ch/tools/protparam.html), the DUX4 protein is considered unstable with an instability index of 71.36.

To confirm a DUX4 stabilization by the proteasome inhibition, we prepared a Western blot with nuclear extracts of proliferating primary myoblasts or myotubes either treated with MG132 or not, and we immunodetected DUX4 with MAb 9A12 (Fig. 5C). Regardless of MG132 treatment, a much stronger 52-kD band was detected in differentiated as compared with proliferating myoblasts. The cultures treated with the proteasome inhibitor presented a stronger DUX4 signal intensity in this experiment. In addition, lower molecular weight bands detected with MAb 9A12 on this Western blot had a decreased intensity upon MG132 treatment, thus showing they resulted from proteolysis (Fig. 5C: red braces).

### The DUX4 protein is expressed in FSHD muscle biopsies

**Fig. 6:**
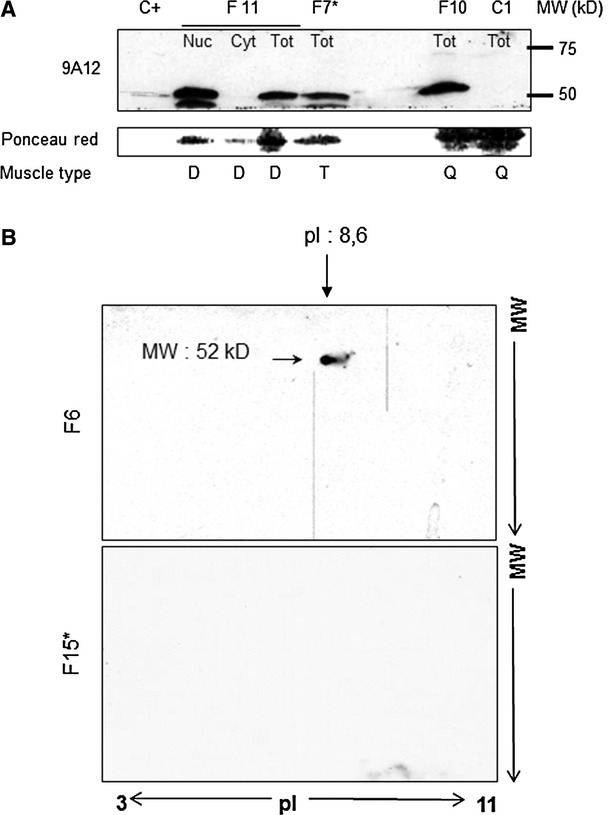
DUX4 expression in facioscapulohumeral muscular dystrophy (FSHD) muscle biopsies. (**A**) Nuclear (Nuc), cytoplasmic (Cyt) or total (Tot) protein extracts from FSHD (F11, F7, F10) or control (C1) muscle biopsies were analysed by 12% PAGE-SDS followed by Western blotting and immunodetection with MAb 9A12. The positive control (C+) is an extract of C2C12 cells transfected with *pCIneo-DUX4*. Ponceau red staining of the membrane was used for loading control. (**B**) Total extract of the F6 and F15 muscle biopsies was analysed by 2D electrophoresis (IEF-PAGE-SDS, see Materials and Methods), followed by Western blotting and an immunodetection with MAb 9A12. The characteristics of these samples are reported in Table S2. (*) FSHD biopsies derived from an affected muscle.

To this date, no report of DUX4 protein detection in FSHD muscle biopsies has been published ([11], reviewed in [20]). This is indeed a technical challenge as the DUX4 protein is unstable and likely expressed in pulses in very few nuclei. For this experiment we used needle biopsies from different muscles (deltoid, trapezius, quadriceps) of two patients with FSHD and a matching control. We prepared Western blots with biopsy protein extracts and could immunodetect the 52-kD band with MAb 9A12 in the FSHD samples but not in the control (Fig. 6A). As expected, DUX4 was observed in total and nuclear but not cytoplasmic extracts. A lower molecular weight band, which most likely corresponded to a DUX4 proteolysis product, was observed and was stronger in the nuclear extracts than in the total extracts probably because of a longer experimental procedure (Fig. 6A). In addition, we characterized the immunodetected protein by 2-D gel electrophoresis (isoelectrofocusing and polyacrylamide-SDS gel) followed by a transfer to a Western blot and immunodetection with MAb 9A12. Owing to the limited material obtained from each biopsy, we used a sample obtained from another patient with FSHD. A single spot was observed at the expected isoelectric point (8.6) and molecular weight (52 kD) of DUX4 (F6 in Fig. 6B) as previously reported for an FSHD myoblast culture [32]. Intriguingly, in the same experimental conditions we could not detect DUX4 in an affected FSHD muscle (F15* in Fig. 6B). As described in the supporting information, DUX4 was detected in two additional FSHD muscle biopsies but not in one control, and in some FSHD surplus obtained from scapular fixation (Figs S6A and B). The characteristics of all the FSHD muscle biopsies tested in this study are shown in Table S2.

## Discussion

### DUX4 is induced during myoblast differentiation

Several groups besides ours have now demonstrated the presence of polyadenylated *DUX4-fl* mRNA in FSHD muscle cells [9–12]. Although we could previously demonstrate DUX4 protein expression in proliferating FSHD but not control myoblasts [9], DUX4 detection is very difficult. Indeed, Snider *et al*. determined that DUX4 was expressed at a relatively abundant amount in only about 1/1000 primary myoblasts [11]. In the present study we confirm a similar low expression for the DUX4 protein by Western blot analysis of proliferating FSHD primary myoblasts. However, in myoblasts grown 4 days in a differentiation medium we detected an increase in DUX4 protein by Western blot analysis (Fig. 5C), which correlated with a higher number of DUX4-positive nuclei (1/200; Fig. 2A and B). Both the DUX4 mRNA ([9, 12]; reviewed in [20]) and protein (the present study) are easier to detect in myotubes than in proliferating myoblasts, suggesting that *DUX4* transcription is induced upon differentiation resulting in both increased expression levels and a larger number of expressing nuclei. DUX4 was detected in cultures derived from both affected and non-affected FSHD muscles, either in non-fused myoblasts or in adjacent nuclei of some myotubes. The number of DUX4-positive nuclei was similar in cultures of both mostly atrophic or disorganized phenotypes, but the staining intensity was stronger in the atrophic type, in keeping with the recently demonstrated role of DUX4 in the atrophic process [19]. Interestingly, we have also detected a weak DUX4 signal in control primary myoblasts, but only upon differentiation. The number of DUX4-positive nuclei was significantly lower than in FSHD myotubes, but we cannot exclude that DUX4 might be expressed normally, in a very limited window, during the myoblast differentiation process.

### The DUX4 expression pattern in FSHD myotubes

**Fig. 7:**
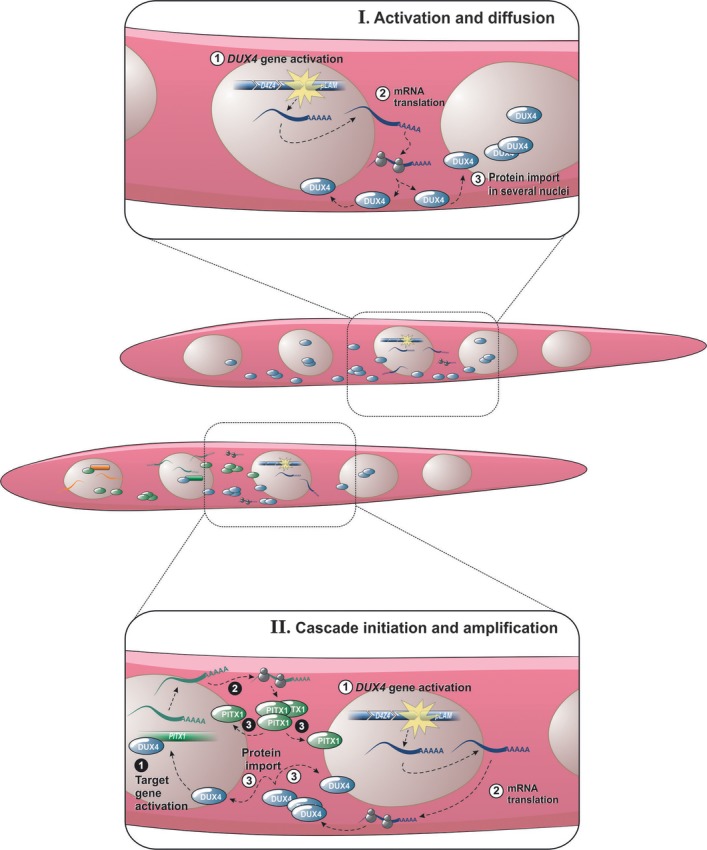
Dynamic model of propagation and initiation of a transcriptional cascade. I: Activation and diffusion. A myotube is a multinucleated cell with a common cytoplasm in which individual nuclei can independently activate gene expression. In an facioscapulohumeral muscular dystrophy (FSHD) myotube, the DUX4 gene is activated in one given nucleus ➀. The DUX4 gene is then transcribed into an mRNA that terminates at the polyadenylation site located in the *pLAM* region. The mRNA is translocated into the cytoplasm domain close to the activated nucleus and it is translated, yielding several molecules of DUX4 protein ➁. The DUX4 protein that carries a nuclear localization signal (NLS) could diffuse in the cytoplasm, and be transported into several neighbouring nuclei ➂. II: Cascade initiation and amplification. In each nucleus that has imported the DUX4 protein, this transcription factor directly activates a number of genes as shown here for the *PITX1* gene ➊. The *PITX1* gene is thus transcribed, its mRNA is translocated into the cytoplasm domain close to the activated nuclei and translated ➋. The molecules of PITX1 protein can diffuse in the cytoplasm and, because they also carry a NLS, they will be imported into more neighbouring nuclei ➌.The transcriptional cascade initiated by DUX4 can further extend because PITX1 is also a transcription factor and targets additional genes such as TP53. At each step of this transcription cascade, the number of activated nuclei and expressed genes increases, causing an amplification of the initial trigger, *i.e*. DUX4 gene activation in a single nucleus. Globally, the DUX4 transcription cascade leads to muscle atrophy, inflammation, oxidative stress and decreased differentiation potential, the key features of FSHD.

In this study, FSHD myotubes presented a DUX4 staining pattern often characterized by one brightly stained nucleus and a progressive decrease in the signal intensity in consecutive nuclei. Similar pictures were independently observed by immunocytochemistry with another antibody, in a very recent publication [33]. This pattern suggests that DUX4 could diffuse to several nuclei in a given myotube and is typical of the limited diffusibility of nuclear proteins in muscle fibres ([25, 27] see Introduction). Indeed, in these multinucleated cells, an individual nucleus could independently express *DUX4* mRNAs that would be exported and translated in the adjacent cytoplasmic domain. The DUX4 protein could then diffuse in the common cytoplasm, and owing to its NLS, it could be imported into several myonuclei in the vicinity of the one that initially transcribed the gene (Fig. 7: Panel I). Such an expression pattern is well known for other muscle nuclear proteins (see Introduction) but is described here for the first time in a pathological context. The initial stochastic *DUX4* gene activation in an isolated nucleus could occur by chromatin remodelling according to different models [34–37]. The DUX4 transcription factor targets a large gene set, leading to increased sensitivity to oxidative stress and myogenic differentiation defects [18]. Some of these genes encode other transcription factors among which we identified the *Pitx1* gene as a direct DUX4 target [9]. This was confirmed by activation of a luciferase reporter gene fused to the human *PITX1* promoter (S. Charron, unpublished data) and more recently by coupled transcriptomic and chromatin immunoprecipitation studies of DUX4-activated genes in human myoblasts [21]. We have observed in the present study that the PITX1 and DUX4 protein expression patterns are similar which is in keeping with their diffusion in consecutive myonuclei. Our data suggest a dynamic model of how the inappropriate DUX4 expression in a limited number of FSHD myonuclei could lead to DUX4 protein diffusion to several nuclei in which it could activate several target genes such as PITX1 (Fig. 7: Panel II). The transcriptional cascade initiated by DUX4 could be further extended because PITX1 is itself a transcription factor that could similarly diffuse to additional nuclei and target additional genes. In several recent publications TP53 [19, 23, 38] as well as the E3 ubiquitin ligases Atrogin-1 and MURF1 [19, 22] which are associated with muscle atrophy were described as parts of the deregulation cascade induced by DUX4. The initial DUX4 trigger in a single nucleus would thus be amplified through a transcriptional cascade that would extend to the whole myotube or myofiber and globally lead to muscle atrophy and inflammation, which are key features of FSHD. This concept is in agreement with a variant of the ‘majority rules’ model recently proposed by Ehrlich and Lacey which involved ‘oscillating non-toxic generation of *DUX4-fl* transcript throughout the FSHD myotube population’ [39].

### The DUX4 and PITX1 proteins half-lifes are regulated by the ubiquitin-proteasome pathway

Our data indicate that, similar to other transcription factors, the DUX4 protein stability appears highly regulated, likely in relation to a role in early development [11]. We found that DUX4 was degraded by the ubiquitin-proteasome pathway (UPP), likely targeting its carboxyl-terminal domain, which contains sequences with destabilization probability (Fig. S5B). Because it is a very potent transcriptional activator [40], very small amounts of DUX4 could be sufficient to initiate the deregulation cascade. We should also mention that although the proteasome usually completely degrades its substrates into small peptides, in a few cases, its proteolytic activity yields biologically active protein fragments as described for several transcription factors (NF-kappa B, Spt23p and Mga2p) [41]. In future studies, it will be interesting to evaluate whether such a proteasomal processing could also occur for members of the FSHD transcriptional cascade, leading to smaller fragments that might exert some biological activity. Finally, our results suggested that other degradation pathways could interfere with DUX4 stability. Indeed, the use of MG132 alone did not always sufficiently stabilize DUX4 to allow its codetection with the product of its *PITX1* target gene. The dynamic expression model presented here, together with an asynchronous regulation of their half-life by the proteasome could explain why the DUX4 and PITX1 proteins were detected either individually in separate nuclei or together in identical nuclei. In the present study, the PITX1 protein was easier to detect in FSHD myotubes than in FSHD muscle biopsies although biopsies had been used to demonstrate its FSHD-specific induction at the mRNA level [9]. We suspect that PITX1 expression is variable, depending on its expression kinetics during myogenic differentiation, the myotube phenotype, the degree of muscle damage and its proteolysis rate.

### The DUX4 protein presents variable subnuclear localization

Our studies indicated different intranuclear distribution patterns for DUX4, *i.e*. punctated or in larger foci. Intriguingly, we have not observed a ring staining as previously described in cells with forced DUX4 expression [17]. The nature and the roles of these nuclear foci still remain to be determined. Like many transcription factors, chromatin proteins, and RNA-processing factors, DUX4 might be compartmentalized and accumulate in distinct nuclear domains that are involved in specific processes (reviewed by [42]). Although the morphology of most nuclei with DUX4-positive foci appeared quite normal, we have observed larger nuclei, some with an irregular outline, and a few fragmented nuclei characteristic of apoptosis (Fig. S4A). However, nuclei expressing the endogenous DUX4 protein in FSHD myoblasts did not exhibit cleaved-PARP staining (Fig. S4B) nor DNA fragmentation (Fig. S4C). Nuclei with the opposite staining pattern (cPARP^+^/DUX4^−^) were increased in FSHD myoblasts and might reflect an apoptotic process initiated by a pulse of DUX4 expression, followed by DUX4 protein degradation, similarly to the explanation we proposed above to explain the presence of PITX1^+^/DUX4^−^ nuclei. Another point to consider is that although DUX4 overexpression induced caspase 3/7 activity and cell death [17, 18], its toxicity was dose dependent. Indeed, a moderate DUX4 expression in C2C12 cells only reduced myogenic differentiation and increased sensitivity to oxidative stress without causing cell death [18]. Further experiments should define whether the presence or shape of DUX4 nuclear foci could be related to the cell cycle or to cellular damage. As described for other transcription factors, nuclear foci could also correspond to sites of target gene transcription or storage [43].

### DUX4 expression in FSHD muscle

Although the *DUX4-fl* mRNA was described by several studies in muscle samples, the DUX4 protein has only been detected in testes, where it is strongly expressed [11]. In the present study, we could for the first time detect the DUX4 protein in FSHD muscle biopsies, particularly in non-affected muscles. In affected muscles, the signal was often at the limit of detection and even missing in a biopsy of a severely affected muscle that presented a major loss of muscle fibres and important fibrosis, suggesting that DUX4 expression is an early event in FSHD.

In conclusion, DUX4 was induced upon differentiation and detected in about 1/200 myonuclei in a panel of FSHD myotubes. Its expression pattern suggested the *DUX4* gene transcription occurred in pulses in rare nuclei followed by a diffusion of the expressed protein to additional nuclei, a mechanism previously described for muscle nuclear proteins in non-pathological contexts. We propose that the DUX4 transcription factor further activates a deregulation cascade in every nucleus to which it has diffused. The transcription factors expressed from some of its target genes such as PITX1 will similarly diffuse to additional nuclei thus further extending the deregulation cascade leading to fibre atrophy or death.

Consistent with a causal role of DUX4 in FSHD, DUX4 is expressed in FSHD muscle, except in a very affected context. Our study opens new perspectives about DUX4 involvement in FSHD, *i.e*. how such a rare protein could cause damages leading to a myopathy. This model should be strengthened in the future by understanding the trigger to *DUX4* transcription pulses, monitoring the DUX4 protein diffusion and deepening our knowledge of the resulting gene deregulation cascade.

## Supporting information

Figure S1. Evaluation of the MAb 9A12 specificity and DUX4 labeling.

Figure S2. Evaluation of MAb 9A12 specificity using DUX4 mRNA silencing.

Figure S3. Immunodetection on western blot of DUX4 expression in FSHD aligned myoblasts and myotubes.

Figure S4. Characteristics of DUX4-positive nuclei.

Figure S5. PEST sequence prediction.

Figure S6. Immunodetection on western blot of DUX4 expression in muscle biopsies.

Table S1. Summary of data about control primary myoblasts.

Table S2. Summary of data about DUX4 expression in FSHD muscle biopsies.
