# Supplementary figures and images for: DUX4 expression in FSHD muscle cells: how could such a rare protein cause a myopathy?
Source: J Cell Mol Med. 2012 Dec 4;17(1):76–89. doi: 10.1111/j.1582-4934.2012.01647.x (PMC3823138; doi:10.1111/j.1582-4934.2012.01647.x)

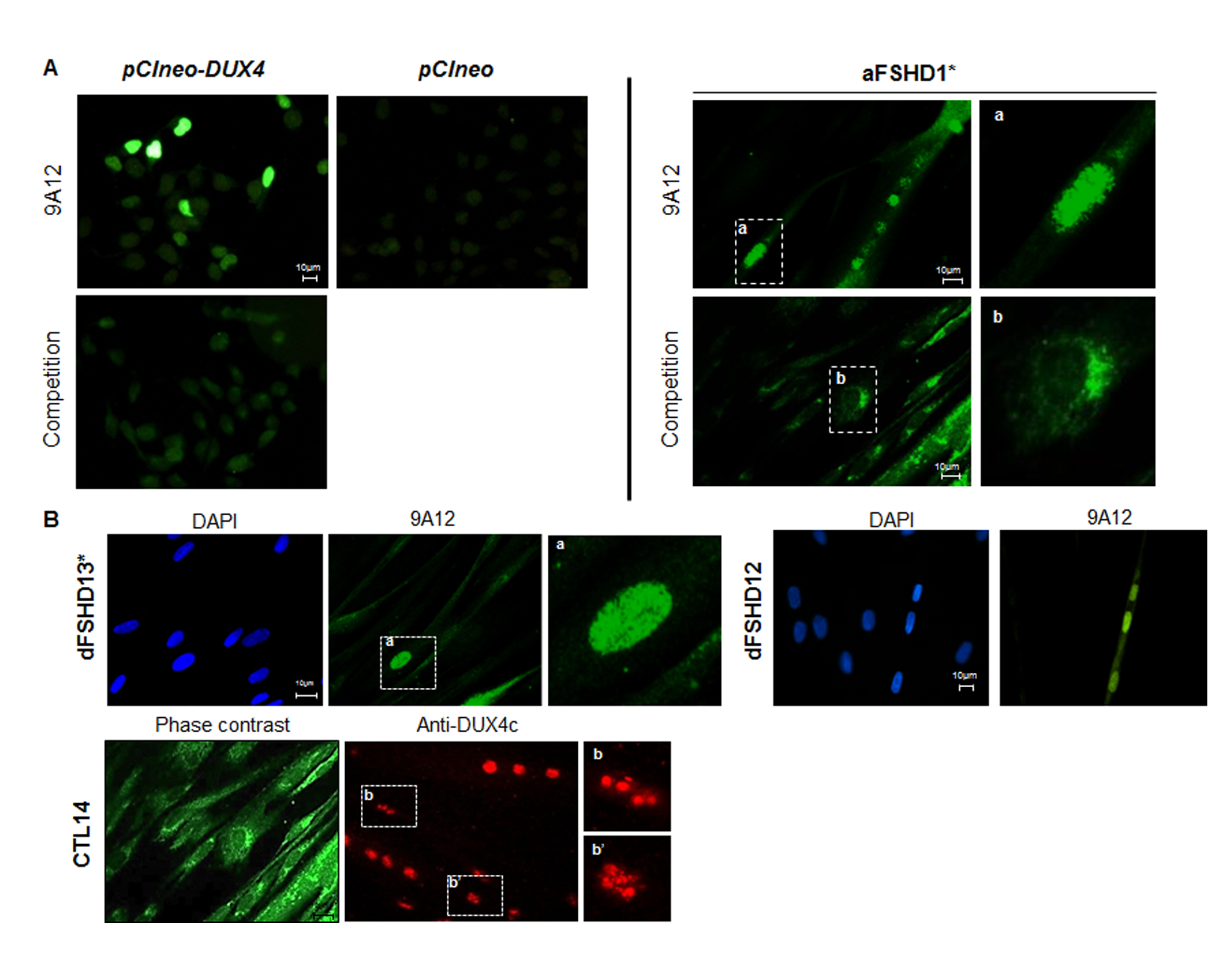

Supplement: Supplementary file 1 [file jcmm0017-0076-SD1.tif]

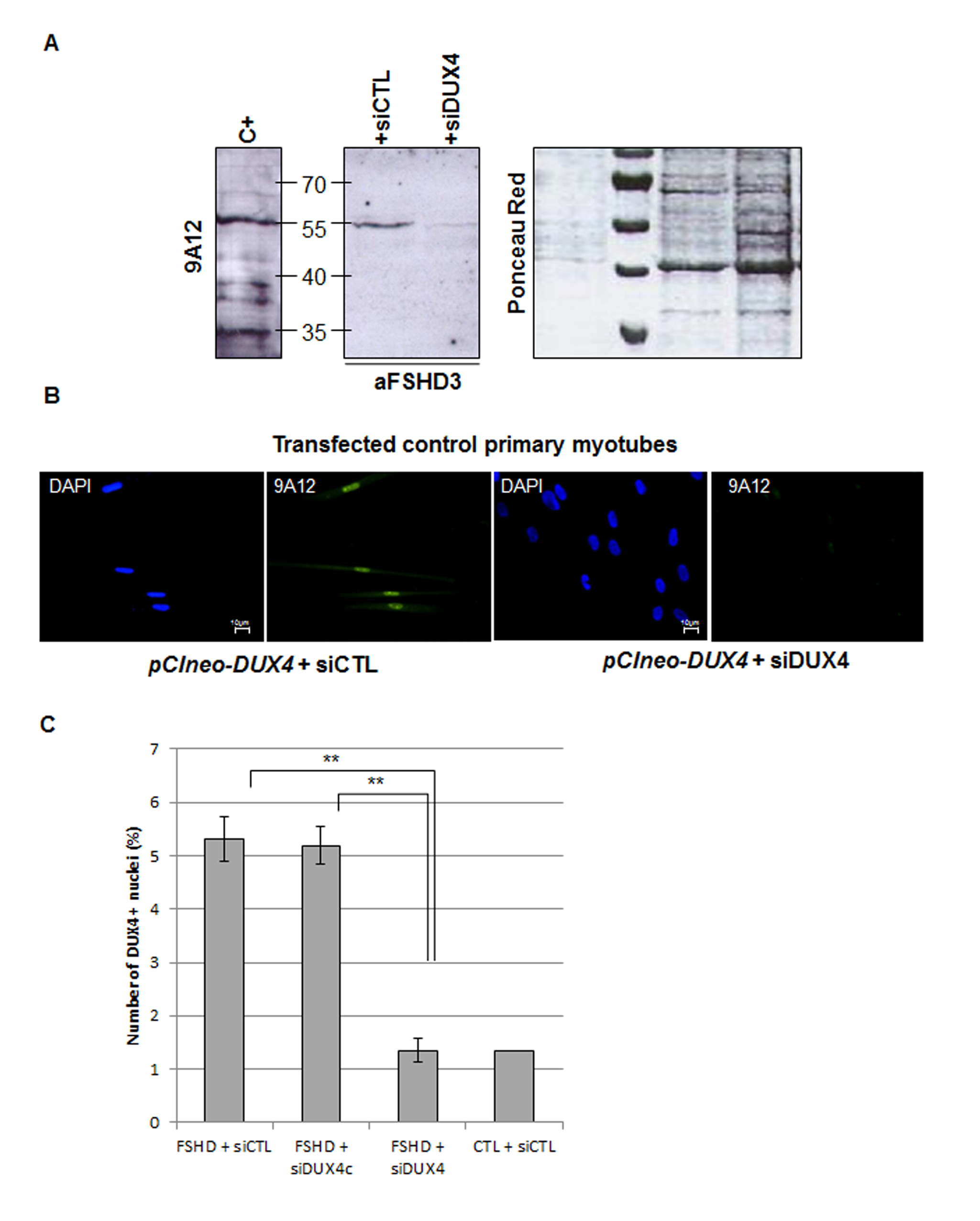

Supplement: Supplementary file 2 [file jcmm0017-0076-SD2.tif]

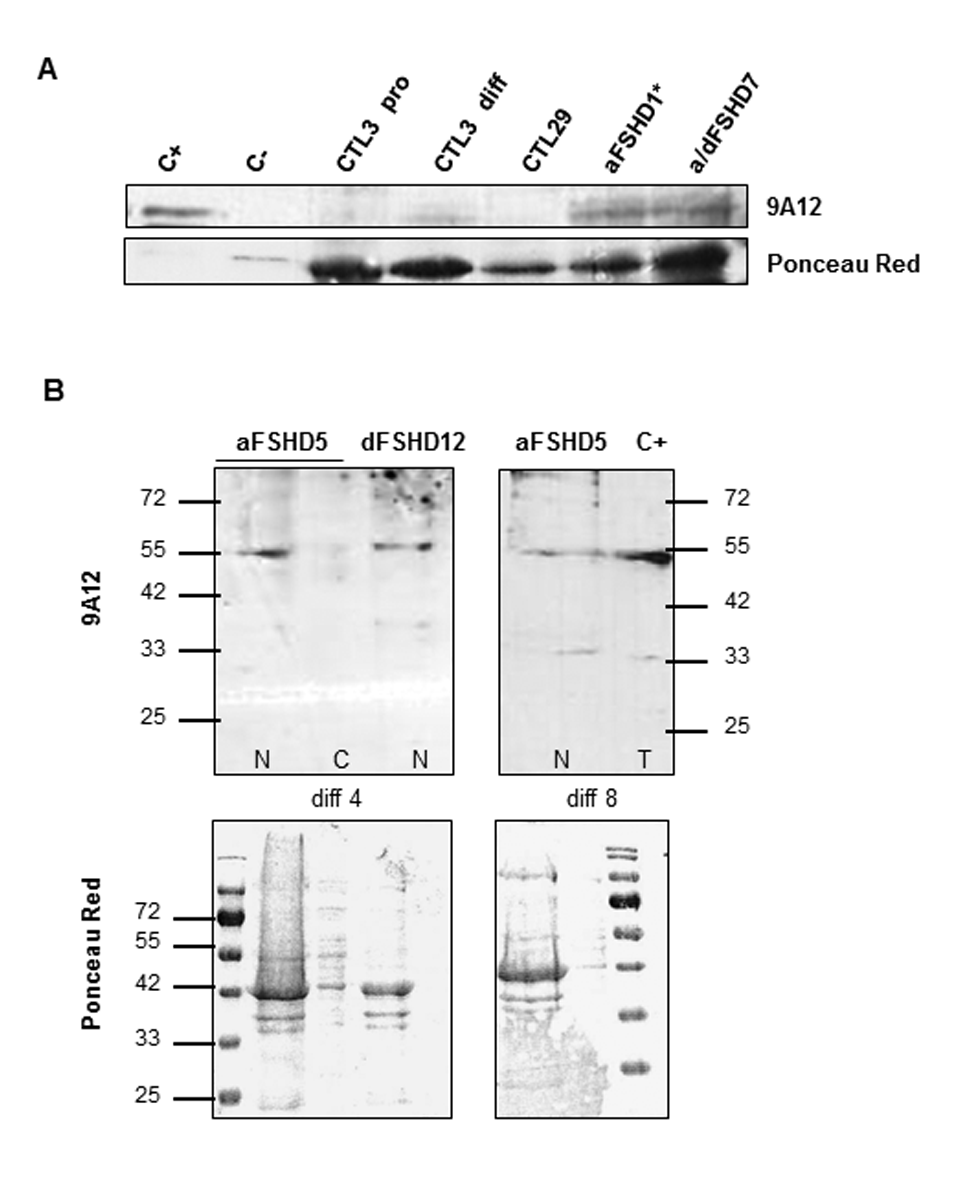

Supplement: Supplementary file 3 [file jcmm0017-0076-SD3.tif]

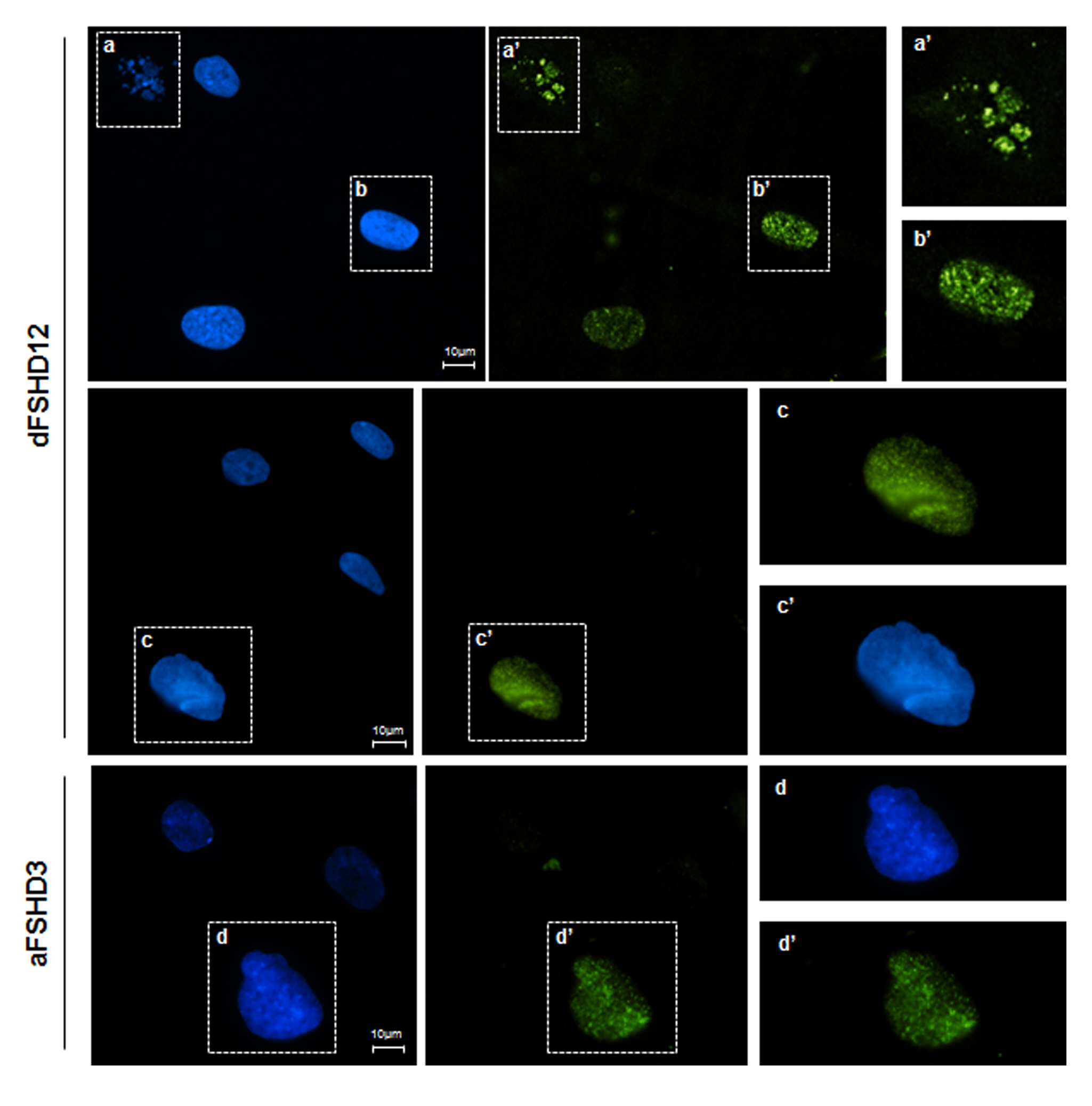

Supplement: Supplementary file 4 [file jcmm0017-0076-SD4.tif]

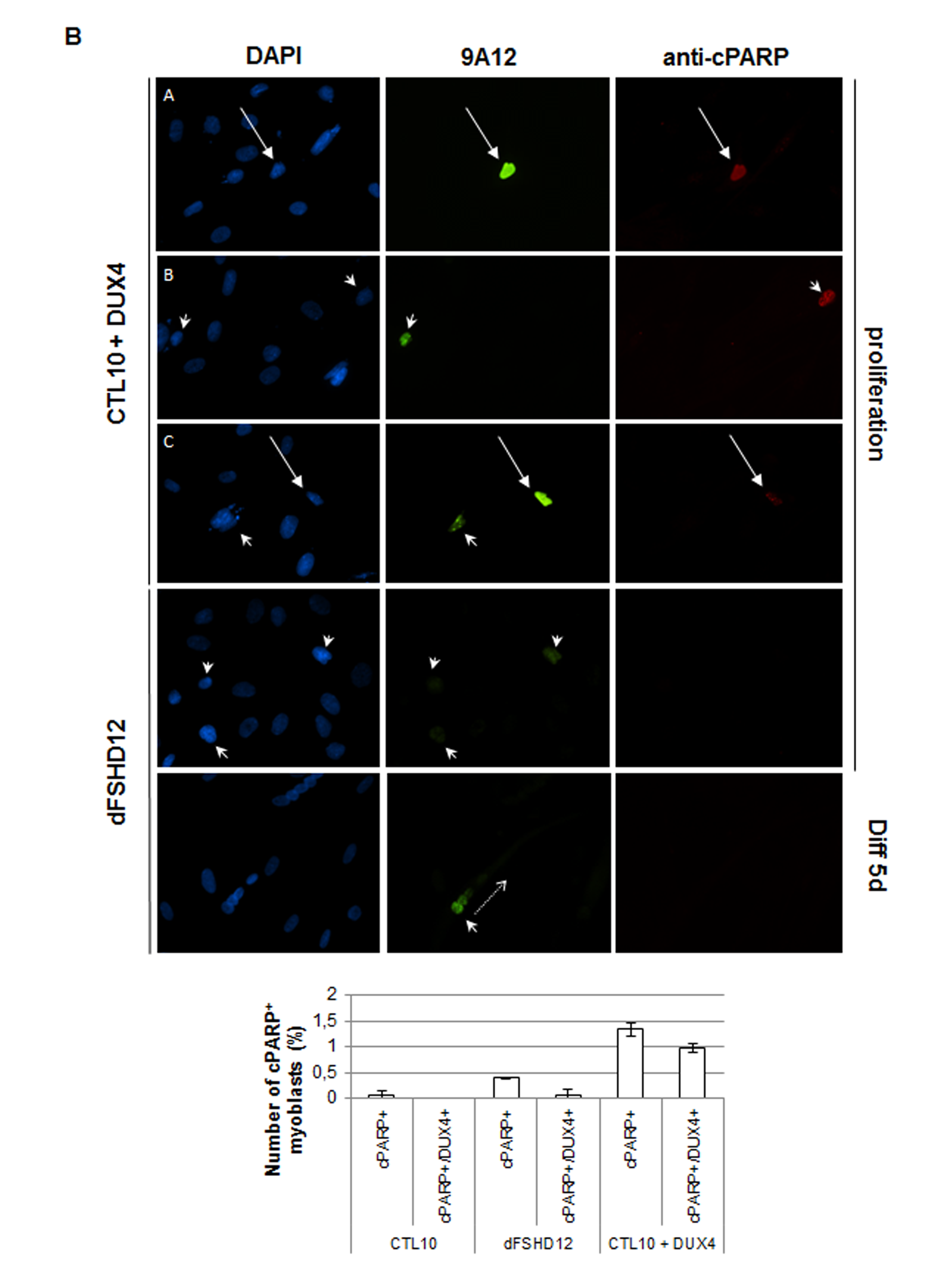

Supplement: Supplementary file 5 [file jcmm0017-0076-SD8.tif]

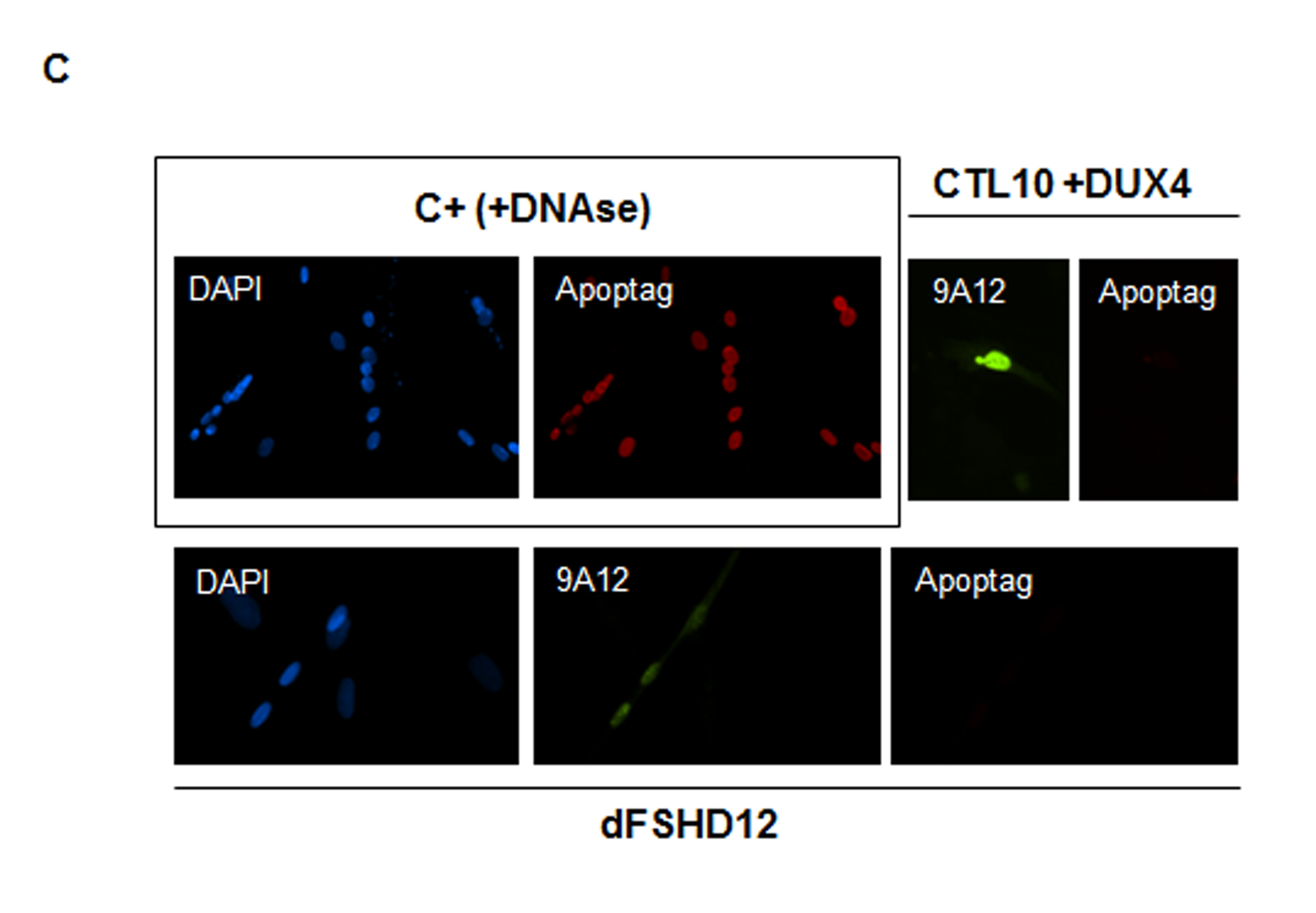

Supplement: Supplementary file 6 [file jcmm0017-0076-SD9.tif]

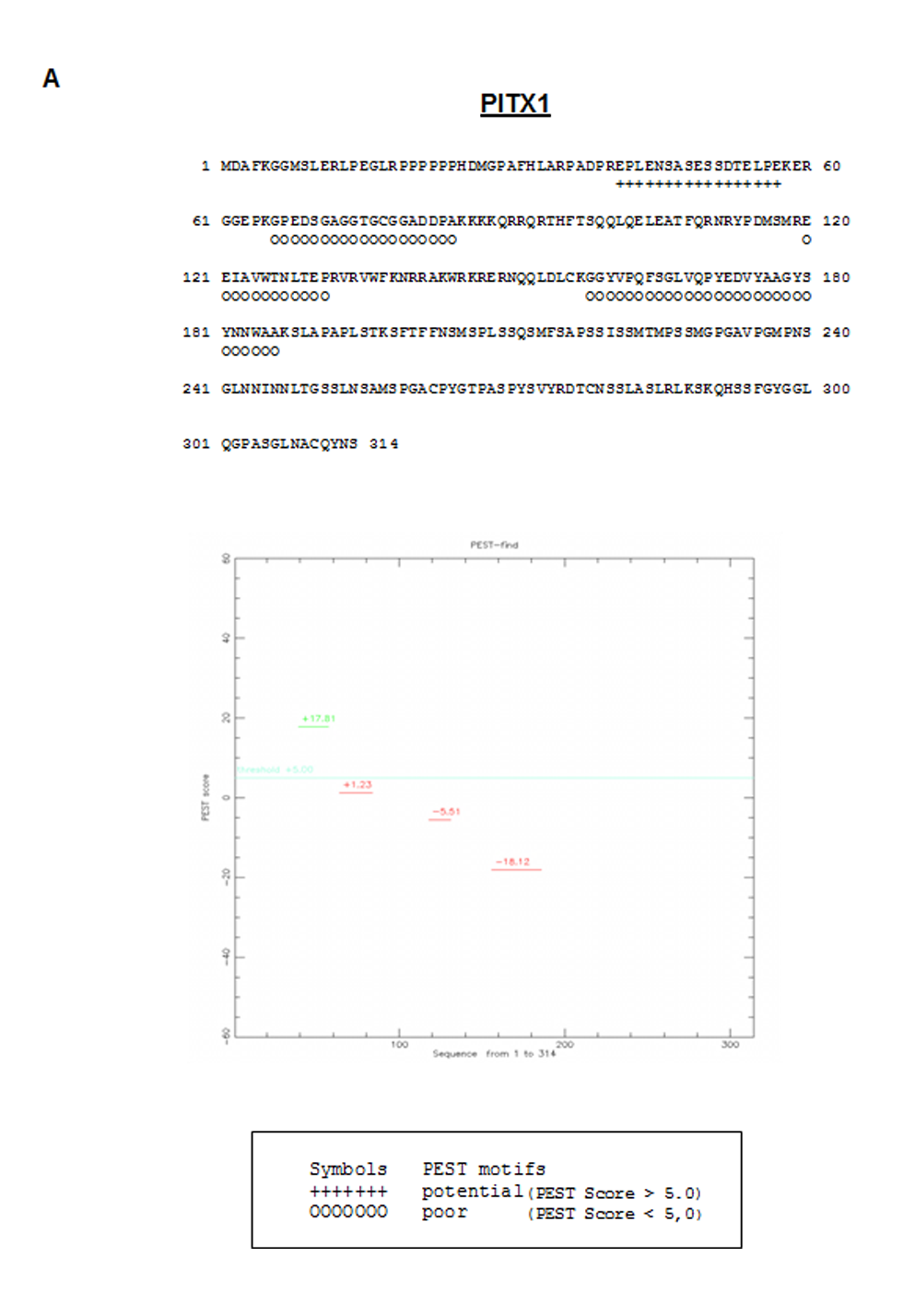

Supplement: Supplementary file 7 [file jcmm0017-0076-SD5.tif]

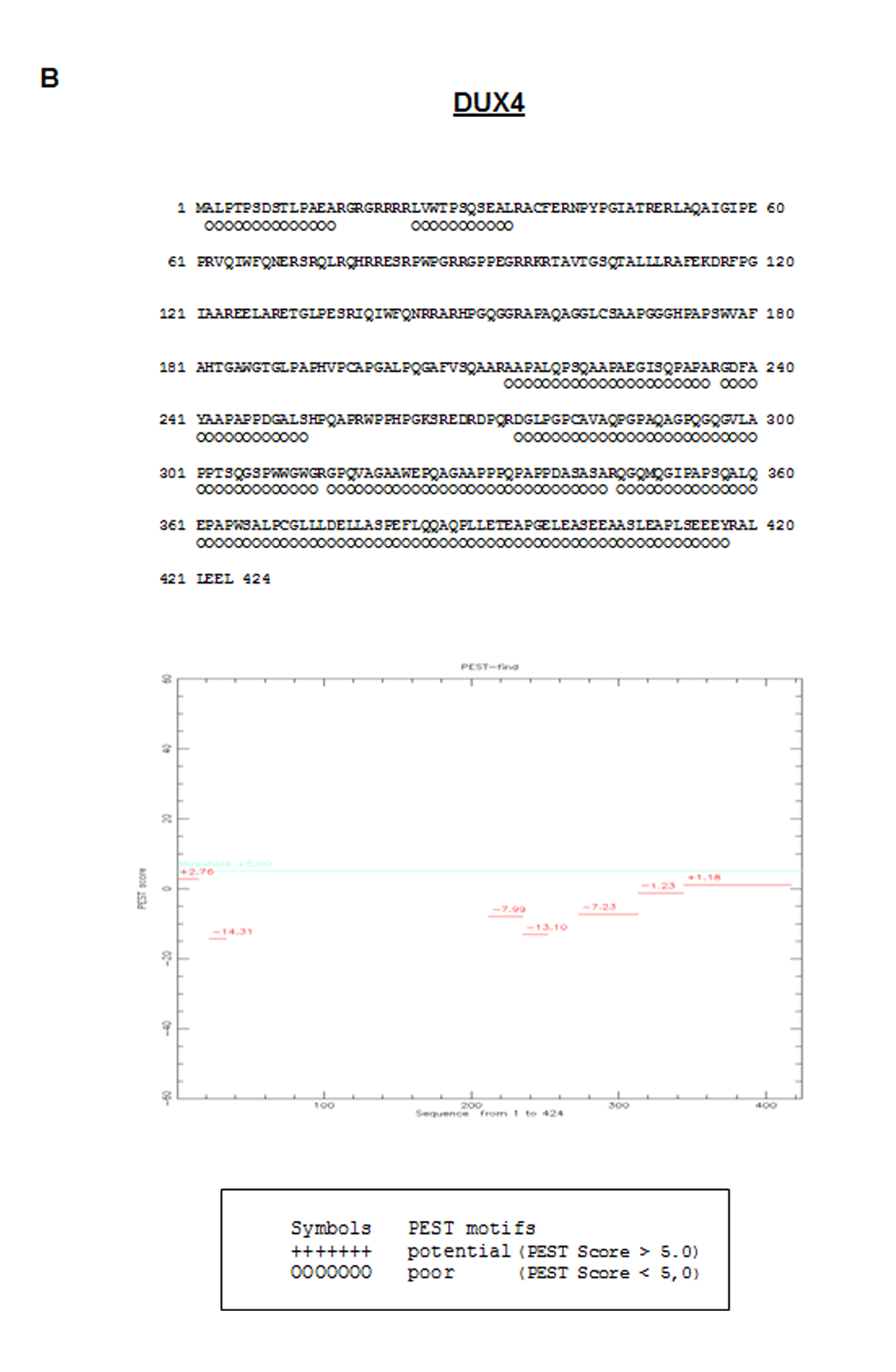

Supplement: Supplementary file 8 [file jcmm0017-0076-SD10.tif]

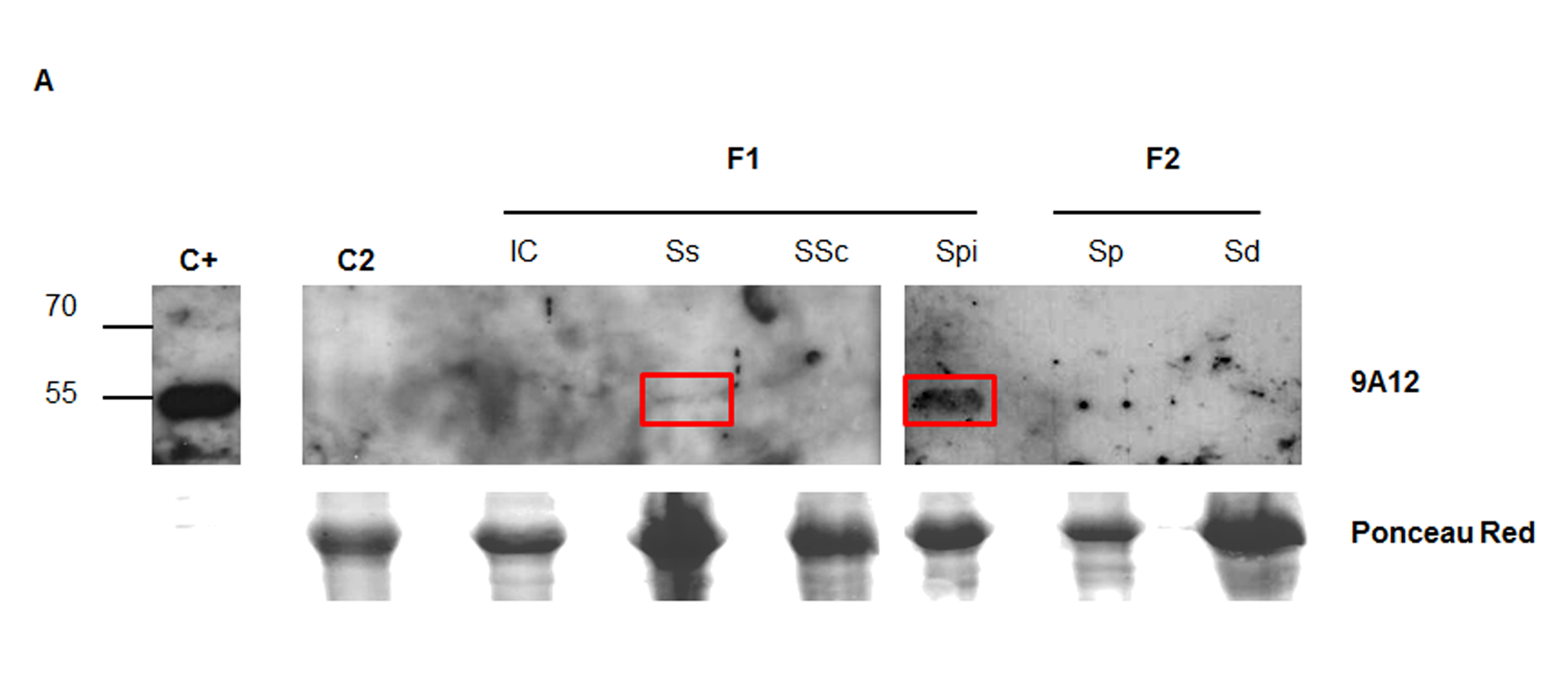

Supplement: Supplementary file 9 [file jcmm0017-0076-SD6.tif]

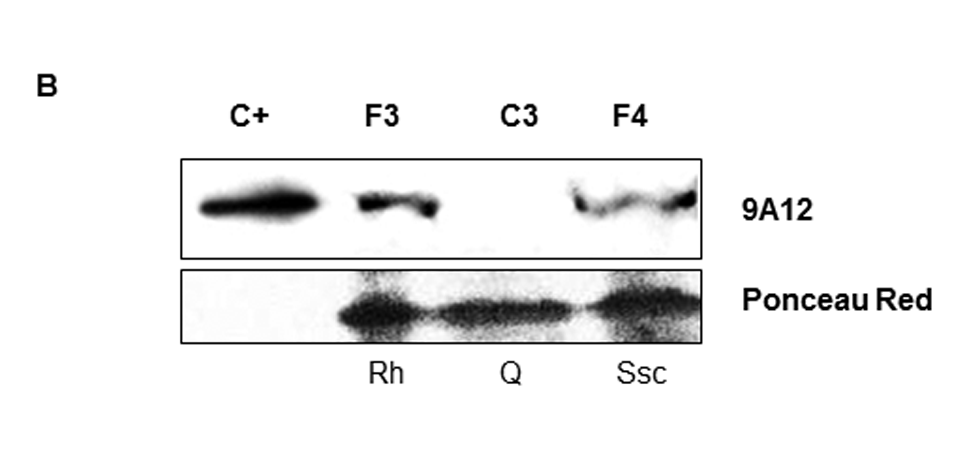

Supplement: Supplementary file 10 [file jcmm0017-0076-SD11.tif]
