# Supplementary material for: DUX4 expression in FSHD muscle cells: how could such a rare protein cause a myopathy?
Source: J Cell Mol Med. 2012 Dec 4;17(1):76–89. doi: 10.1111/j.1582-4934.2012.01647.x (PMC3823138; doi:10.1111/j.1582-4934.2012.01647.x)
Supplement: Supplementary file 11 [file jcmm0017-0076-SD7.docx]

**Supporting informations**

**SI text: MAb 9A12 specificity against DUX4**

We have previously shown that MAb 9A12 specifically detected both DUX4 and DUX4c on Western blots prepared with extracts of TE671 cells transfected with *pCIneo-DUX4* or-*DUX4c* [1]. When immunofluorescence was performed with MAb 9A12 on TE671 cells transfected with *pCIneo-DUX4,* the nuclear signal disappeared upon competition with a 10-fold excess of the antigenic recombinant protein (**Fig. S1A,** left panel). Similarly, MAb 9A12 gave a nuclear signal in primary FSHD myotubes that was lost upon competition with the antigenic protein, but the cytoplasmic background remained, indicating that the nuclear staining was specific (**Fig. S1A,** right panel). Intriguingly, MAb 9A12 only detected DUX4c when this protein was over-expressed in cells transfected with the strong *pCIneo-DUX4c* expression vector [1]. In either condition and in **Fig. S3B**, no 47 kDa-DUX4c band was detected in the Western blot below the 52 kDa-DUX4 one. MAb 9A12 did not react with the endogenous DUX4c protein in primary human myoblasts/myotubes. Thus in primary FSHD myotubes MAb 9A12 only detects the endogenous DUX4 protein. This specificity was confirmed using an siRNA directed against the *pLAM* region found in the *DUX4* mRNA but missing in the *DUX4c* mRNA [2]. In this experiment, a Western blot was prepared with nuclear extracts of FSHD primary myotubes treated with a scrambled siRNA or the siRNA against *DUX4*: upon immunodetection with MAb 9A12, the DUX4 protein was strongly decreased in cells treated with the *DUX4-siRNA* (**Fig. S2A**).

Additional evidences that MAb 9A12 doesn’t react with the myoblast endogenous DUX4c are experiments done with a rabbit antiserum directed against a DUX4c-specific peptide (residues 350 to 365; [3]). This antiserum recognized a large number of nuclei (**Fig. S1B**) in FSHD as well as in control myoblasts, both in proliferation and differentiation [3]. In contrast, MAb 9A12 stained very few control myonuclei and only after 4 days of differentiation (**Figs. 2-4**) and only about 5% of nuclei in FSHD myotubes (**Figs. 2-4, S1B**): in elongated myoblasts that are not yet part of a myotube, we observed a DUX4 labeling either in foci (**Fig. 3A and B**) or as a dense punctated pattern covering the entire nucleus (**Figs. S1B: a**). A similar labeling was observed in myotube nuclei; the staining intensity was strong in a few myonuclei and progressively decreased in the neighboring ones (**Figs. 3D, 4D, S1A** right panel**; S1B,** right panel). The immunofluorescence signal generally overlapped with regions of intense DAPI staining (**Fig. S4**). Finally, the number of nuclei stained by MAb 9A12 was not modified by a *DUX4c-siRNA* but significantly reduced after treatment with a *DUX4-siRNA* (**Fig. S2B and C**), underscoring the fact that this corresponded to a DUX4-specific signal. The immunofluorescence staining corresponding to either DUX4c or DUX4 can thus easily be discriminated by using either the rabbit serum directed against a DUX4c-specific peptide or MAb 9A12, respectively.

**SI Tables**

| Code | Age | Sex | Muscle Type | Differentiation stage (Method, Figures) |
| --- | --- | --- | --- | --- |
| CTL3* | 41 | M | Q | Aligned myoblasts and diff 6 (WB, Fig.S3A) |
| CTL6* | 23 | F | P | diff 4 (IF, Fig.4F) |
| CTL10* | 21 | M | Q | diff 5 (IF, Fig.3) |
| CTL12* | 35 | F | Q | diff 4 (IF, Fig.2A) |
| CTL14* | 43 | M | Q | diff 6 (IF, Fig.S1B) |
| CTL29^#^ | 23 | M | Q | Aligned myoblasts (WB, Fig.S3A) |
| * characterized in Barro et al., 2010 ^#^ provided by Dr. D. Figlewicz | | | | |

**Table S1: Summary of data about control primary myoblasts.**

Name of the cell line; age and sex of the healthy volunteer (M: male; F: female); site of the muscle biopsy (Q=*quadriceps*; P=*paravertebral*); differentiation stage and DUX4 detection method used (WB: immunodetection on western blot, IF: immunofluorescence). The informations about control individuals CTL3, 6, 10, 12 and 14 were previously given in [4]. The CTL29 sample was kindly provided by Dr. D. Figlewicz.

| Code | References | Age | Sex | D4Z4 units | Muscle type (*affected) | DUX4 expression (Method) |
| --- | --- | --- | --- | --- | --- | --- |
| F1 |  | 43 | M | 5 | IC, Ss, Ssc, Spi | -, +/-, -, + (WB, Fig.S6) |
| F2 |  | 35/40 | F | 5 | Sp, Sd | -, - (WB, Fig.S6) |
| F3 |  | 31 | M | 4.8 | Rh | + (WB, Fig. S6) |
| F4 |  | 40 | M | 5 | Ssc | + (WB, Fig. S6) |
| F6 | Primary myoblasts described in Barro et al., 2010 | 23 | F | 8 | Q | + (2D: Fig.6B) |
| F7 | Biopsy analysed for DUX4c expression in Ansseau et al., 2009 | 31 | M | 5 | T* | + (WB: Fig.6A) |
| F10 | Biopsy analysed for DUX4c expression in Ansseau et al., 2009 | 53 | F | 7+5 | Q | + (WB: Fig.6A) |
| F11 |  | 31 | M | 5 | D | + (WB: Fig.6A) |
| F15 | Primary myoblasts described in Barro et al., 2010 | 52 | F | 8 | Q* | - (2D: Fig.6B) |
| * biopsy derived from affected muscle | | | | | | |

**Table S2: Summary of data about DUX4 expression in FSHD muscle biopsies.** Code for FSHD biopsies; publications in which the same biopsy or derived primary myoblast line were previously analysed (DUX4c expression: [3]; morphological characterization of primary myotubes : [4]); age and sex of the patient (M: male; F: female); number of D4Z4 units; muscle site of the biopsy (IC: *intercostales*, Ss: *seratus superior*, SSc : *subscapularis*, Spi: *spinalis,* Sp: *serratus anterior* (proximal), Sd: *serratus anterior* (distal), Rh: *Rhomboideus*, Q: *quadriceps* (*vastus lateralis*), T: *trapezius*, D: *deltoid*, *: affected muscles); approximate DUX4 expression level (- : no expression, + : expression, +/- : weak expression) and used method.

**SI Figure legends**

**Figure S1: Evaluation of the MAb 9A12 specificity and DUX4 labeling**. (**A**) Left panel: TE671 cells were seeded on coverslips in 6-well plates and transfected 24h later with 1µg of the indicated plasmids (Fugene 6, Roche). After 24h, the cells were fixed in 4% paraformaldehyde. Immunostaining was performed by standard procedures with MAb 9A12 (1:50) and anti-mouse IgG secondary antibodies coupled to FITC (1:100, Dako, Amersham Biosciences). MAb 9A12 recognizes the DUX4 protein (green) in cells transfected with the *pCIneo-DUX4* expression vector*.* The labeling was weakened when MAb 9A12 was pre-incubated with a 10-fold excess of the antigen (competition). Cells transfected with the insertless *pCIneo* vector were used as a negative control. Right panel: the immunostaining was performed as above on aFSHD1 myotubes at 4 days of differentiation. **(B)** Top panels: DUX4 (green) was detected by immunofluorescence in the nuclei of dFSHD13 and dFSHD12 myotubes 4 days after the induction of differentiation. a: enlarged field of the left box. DAPI (blue) was used to visualize nuclei. Lower panel : DUX4c (red) was detected by immunofluorescence with a specific rabbit antiserum in nuclei of control (CTL14) myotubes, 6 days after inducing differentiation. b and b’: enlarged fields of the left boxes. Sub-nuclear foci are detected. Phase contrast microscopy was used to visualize myotube morphology and the nuclei position.

**Figure S2: Evaluation of** **MAb 9A12 specificity using DUX4 mRNA silencing. (A)** FSHD primary myoblasts (aFSHD3) were transfected either with a control scrambled siRNA (siCTL, 30 nM) or *DUX4-siRNA3* (siDUX4, 10 nM, [2]) using a reverse-transfection method (*siPORTneoFX* reagent, Ambion). The *DUX4-siRNA* targets mRNA sequences in the 3'UTR region transcribed from *pLAM* to avoid cross reactivity with the highly similar *DUX4c* mRNA as described in [2]. Differentiation was induced 4h after transfection, and the cells were harvested 3 days later. Nuclear proteins were extracted and 20µg analyzed by 12% PAGE-SDS and western blotting followed by immunodetection with MAb 9A12 (see Material and Methods). The positive control (C+) corresponds to TE671 cells transfected with *pCIneo-DUX4* (5µg). Ponceau red staining of the membrane was used as a loading control. **(B)** DUX4 (green) was detected by immunofluorescence with MAb 9A12 in immortalized control myoblasts transfected with *pCIneo-DUX4* and differentiated during 8 days. This staining strongly decreased when the myoblasts were treated with the *DUX4-siRNA*. DAPI staining (blue) was used to localize the nuclei**. (C)** Quantification of DUX4-positive nuclei in FSHD myotubes transfected with either the control siRNA (siCTL), or *DUX4c-siRNA* (siDUX4c) or *DUX4-siRNA* (siDUX4) compared to control myotubes transfected with the control siRNA (CTL + siCTL). The number of DUX4-positive nuclei was determined in 30 random fields for each cell culture. The percentage was calculated relatively to the number of DAPI-positive nuclei and the histogram represents the percentage means. The significance was evaluated by an ANOVA test. **p<0.01 was considered significant.

**Figure S3: Immunodetection on western blot of DUX4 expression in FSHD aligned myoblasts and myotubes. (A)** Total protein extracts from FSHD (aFSHD1 and a/dFSHD7) or control (CTL29 and CTL3) primary myoblasts were separated by 12% PAGE-SDS, transferred to a Western blot and DUX4 was immunodetected with MAb 9A12 as described in Materials and Methods. Total extracts were prepared from confluent and aligned myoblasts, except for the CTL3 sample that was also analyzed 6 days after the induction of differentiation in 2% horse serum (HS). Total extracts of TE671 cells transfected with *pCIneo-DUX4* were used as a positive control (C+) and cells transfected with the *pCIneo* empty vector as a negative control (C-). Ponceau red staining of the membrane was used for loading control. **(B)** Nuclear (N), cytoplasmic (C) or total (T) protein extracts from FSHD (aFSHD5 and dFSHD12) myotubes were analyzed by 12% PAGE-SDS, Western blotting and immunodetection with MAb 9A12, 4 (diff 4) or 8 (diff 8) days after the induction of differentiation. Total extracts of TE671 cells transfected with *pCIneo-DUX4* were used as a positive control (C+). Ponceau red staining of the membrane was used for loading control.

**Figure S4: Characteristics of DUX4-positive nuclei. (A)** DUX4 (green) was detected by immunofluorescence in the nuclei of FSHD myotubes (aFSHD3, dFSHD12) 4 days after the induction of differentiation. a-d, a’-d’ correspond to enlarged fields from the left boxes. DAPI (blue) was used to visualize nuclei. Although the morphology of DUX4-positive nuclei was often unchanged (b, b’), some of them were fragmented (a, a’) or larger with an irregular nuclear membrane (c, c’, d, d’). The DUX4-positive nuclei also presented a stronger DAPI staining (b-d). **(B)** To assess whether these characteristics could reflect a DUX4-dependent apoptotic process, we used a rabbit polyclonal antibody against cleaved PARP (cPARP) detecting the large fragment of human PARP (poly ADP-ribose polymerase, Cell Signaling, 1/200, incubation O/N at 4°C) as described in [5]. The cleavage of PARP is considered indicative of functional caspase activation during apoptosis. DUX4 (green) and cPARP (red) were detected by immunofluorescence in the nuclei of some control primary myoblasts transfected with *pCIneo-DUX4* (CTL10+DUX4)*.* However, cPARP was only detected in very few FSHD (dFSHD12) myoblasts and myotubes (diff5d: 5 days after the induction of differentiation) nuclei. Those rare cPARP-positive nuclei were not co-localized with endogenous DUX4 staining. The bottom panel represents the number of PARP-positive nuclei determined in 20 random fields for each cell culture (replicates). The number of nuclei with a cPARP/DUX4 co-localization is also indicated. The percentage was calculated relatively to the number of DAPI-positive nuclei and the histogram represents the percentage means. **(C)** To assess DNA fragmentation in DUX4-positive nuclei on the same samples, we used the Apoptag Red *In Situ* Apoptosis Detection kit (Millipore, MA, USA) according to the manufacturer’s instructions (Red) followed by an immunofluorescence with MAb 9A12 (green). DUX4-positive nuclei were not stained for fragmented DNA. As a positive control (C+), fixed and permeabilized myoblasts were treated with Benzonase nuclease (Novagen, 10U/µl) in Tris 30mM pH7.2/MgCl2 4mM/DTT 0.1mM.

**Figure S5: PEST sequence prediction.** The PESTfind software (http://emboss.bioinformatics.nl/cgi-bin/emboss/epestfind) was used to predict PEST sites within the PITX1 (A) and the DUX4 (B) protein sequences. The upper panel gives the protein sequences and the localization of potential (score >5.0) or poor PEST motives (score<5.0) as indicated. The PEST score is a combination of local enrichment terms (of critical amino acids: D, E, P, S and T) and hydrophobicity. As illustrated in the lower panel, valid PEST motifs below the threshold score (5.0) are considered by the software as poor (red), whereas PEST scores above the threshold score are called potential PEST motives (green).

**Figure S6: Immunodetection on western blot of DUX4 expression in muscle biopsies. (A)** Total extracts from FSHD (F1 and F2), or control (C2) muscle biopsies were analyzed as described in **Fig. 6**. The FSHD muscle types are indicated (IC: *intercostales,* Ss: serratus *superior*, SSc : *subscapularis,* Spi: *spinalis*, Sp: *serratus anterior* (proximal), Sd: *serratus anterior* (distal)). **(B)** Total extracts from FSHD (F3 and F4), or control (C3) muscle biopsies were analyzed as described in **Fig. 6**. The FSHD muscle types are indicated (Rh: *rhomboideus,* Q: *quadriceps* (*vastus lateralis*), SSc : *subscapularis*).

**References**

1. **Dixit M, Ansseau E, Tassin A**, ***et al.*** DUX4, a candidate gene of facioscapulohumeral muscular dystrophy, encodes a transcriptional activator of PITX1. *Proc Natl Acad Sci USA*. 2007; 104: 18157–62.

2. **Vanderplanck C, Ansseau E, Charron S**, ***et al.*** The FSHD Atrophic Myotube Phenotype Is Caused by DUX4 Expression. *PLoS ONE.* 2011; 6: e26820.

3. **Ansseau E, Laoudj-Chenivesse D, Marcowycz** **A,** ***et al.*** DUX4c is up-regulated in FSHD. It induces the MYF5 protein and human myoblast proliferation. *PLoS ONE.* 2009; 4: e7482.

4. **Barro M, Carnac G, Flavier S**, ***et al.*** Myoblasts from affected and non-affected FSHD muscles exhibit morphological differentiation defects. *J Cell Mol Med.* 2010; 14: 275–89.

5. **Bressenot A, Marchal S, Bezdetnaya L,** ***et al.*** Assessment of apoptosis by immunohistochemistry to active caspase-3, active caspase-7, or cleaved PARP in monolayer cells and spheroid and subcutaneous xenografts of human carcinoma. *J Histochem Cytochem.* 2009; 57: 289–300.
